# Modeling Calcite’s Sensitivity to Biogenic CO_2_ Production: A Pathway to Soil CO_2_ Efflux Partitioning

**DOI:** 10.1021/acs.est.5c07428

**Published:** 2025-10-30

**Authors:** Kenneth Tetteh, Georg Guggenberger, Leopold Sauheitl, Kazem Zamanian

**Affiliations:** Institute of Earth System Sciences, Section Soil Science, 598001 Leibniz University Hannover, Herrenhäuser Straße 2, 30419 Hannover, Germany

**Keywords:** soil inorganic carbon, carbonate reactivity, biogenic CO_2_, δ^13^C tracing, residue decomposition, bicarbonate export, optode technology, reactive transport modeling, inorganic CO_2_

## Abstract

Soil inorganic carbon (SIC), primarily calcite, represents a potentially reactive carbon reservoir, influencing soil–atmosphere CO_2_ exchange and acid–base buffering processes. Though often considered stable, SIC is sensitive to biogenic CO_2_ and acidification, risking extra CO_2_ emissions beyond soil organic matter (SOM) mineralization. This study investigates SIC reactivity using δ^13^C-enriched calcite (11.9 t ha^–1^, +102.02‰) under organic residue decomposition, examining the effects of residue type (maize vs wheat), degradability (leaves vs roots), and placement (mixing vs mulching). Incubations at 25 °C with 80% soil–water saturation coupled high-resolution pH optodes and HYDRUS-PHREEQC simulations to quantify SIC reactivity. Mixed applications of labile maize leaves (C:N = 17.3) intensified topsoil (∼50% of the 10 cm column) acid loading (pH 7.9 → 5.7), promoting decarbonation and deepening acidification front (>3.2 cm). Soil respiration emerged as a key influencer of CO_2_ pressures, controlling porewater acid carrying capacity. Dissolution promoters (H_2_O, H^+^, and H_2_CO_3_) drove topsoil decarbonation (0.84 t C ha^–1^ in mixed profiles vs 0.06 t C ha^–1^ in mulched) and subsoil (5–10 cm) bicarbonate accrual. δ^13^C tracing showed SIC-sourced CO_2_ peaks (+25 to +51‰, 40–60% contribution) during incubation’s first quarter (∼day 16–24) prior to SOM-domination (0 to −12‰, 20–10%), reflecting a mixed continuum of CO_2_ sources, SC turnover, and climate feedbacks.

## Graphical abstract

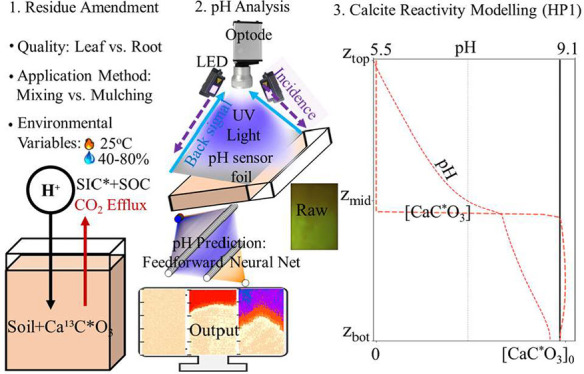

## 1 Introduction

Real-time environmental monitoring technologies are reshaping our understanding of soil–atmosphere interactions and biogeochemical processes. Among these, planar optode imaging enables high-resolution, noninvasive mapping of spatial-temporal pH dynamics at the microscale, capturing the chemical gradients that regulate microbial activity, nutrient availability, and carbon turnover.^1–3^ In parallel, reactive transport models such as HYDRUS-PHREEQC (HP1) simulate coupled geochemical processes (including pH shifts and carbonate (de)­dissolution) under variable soil CO_2_ conditions with high fidelity.^4–6^ Integrating these approaches allows for the quantitative assessment of how biological acidification and mineral buffering govern soil–atmosphere CO_2_ exchange under changing environmental conditions.

In the context of climate change, soils store more than three times the carbon found in vegetation and the atmosphere combined,^7^ making them a critical component of the global carbon cycle. While soil organic carbon (SOC) has been extensively studied, soil inorganic carbon (SIC), comprising dissolved inorganic carbon (DIC) species and solid-phase carbonates such as calcite, remains underexplored, despite its substantial global reservoir (∼950–1400 Pg C in the top meter of soil).^8,9^

SIC plays a dual role: it can either sequester or emit CO_2_ depending on environmental conditions.^10^ The dissolution and reprecipitation of carbonates are tightly coupled to the partial pressure of CO_2_ (pCO_2_), which fluctuates with root respiration, microbial activity, and organic residue decomposition.^11,12^ These biogenic processes locally increase CO_2_ concentrations, intensifying proton generation and lowering the pH, which in turn promotes carbonate dissolution. This feedback influences soil buffering capacity, cation availability, and the balance between inorganic and organic carbon pools.^13,14^

Despite its significance, the vulnerability of SIC to biogenic CO_2_ produced during residue decomposition remains unclear. In agricultural and managed systems, where residue inputs and microbial respiration are high, elevated pCO_2_ and acid production may accelerate carbonate dissolution and CO_2_ release.^15–24^ Beyond the partial compensation of SIC-derived CO_2_ emissions through subsequent carbonate reprecipitation at greater depths,^6^ topsoil decarbonation caused by carbonate depletion can disrupt SOM–mineral associations and weaken SOC stabilization mechanisms, thereby enhancing CO_2_ emissions from SOC mineralization.^10–14^ Yet, mechanistic quantification of this process (particularly linking residue quality, placement, and acidification depth to SIC turnover) has been lacking.

This study addresses this knowledge gap by integrating high-resolution planar optode imaging with HP1 simulations to investigate how biogenic CO_2_ from residue decomposition drives SIC reactivity and acidification. Specifically, we assess how residue type (maize vs wheat), degradability (leaves vs roots), and placement (mixing vs mulching) influence carbonate dissolution, CO_2_ efflux partitioning, and bicarbonate redistribution. The results advance understanding of the interplay between organic and inorganic carbon pools, offering new insights for soil carbon accounting and climate feedback prediction.

We tested three hypotheses under controlled conditions (25 °C and 80% soil–water saturation):

Residue decomposition elevates soil CO_2_ pressures and acidifies the rhizosphere, creating spatial and temporal pH gradients that are strongest at residue–soil contact zones.
Residue quality and placement regulate acid propagation and calcite dissolution, with mixed applications, particularly of labile leaf residues, enhancing substrate contact, proton mobility, and dissolution depth compared to mulching.
Applied δ^13^C-enriched calcite contributes substantially to early CO_2_ efflux, but its influence declines over time as equilibrium is reached and SOM-derived CO_2_ becomes dominant.

## 2 Materials and Methods

### 2.1 Soil, Layout, and Sampling

#### 2.1.1 Soil and Layout

Carbonate-free topsoil (0–20 cm) was collected from a Cambisol developed on loess in Steinbruch Nußloch, Germany (49°18′48″N and 8°42′38″E), a temperate oceanic site (mean annual rainfall: 624 mm; mean temperature: 9.5 °C). The non-arable site supports sparse woody vegetation and mixed perennial C_3_ grasses, with no mineral fertilization or liming for at least the past decade, minimizing anthropogenic carbon inputs and preserving natural soil inorganic carbon (SIC) dynamics.

The silty loam soil (Table S1) was mixed with ^13^C-enriched CaCO_3_ powder (δ^13^C = 102.02‰) at a ratio of 99:1 (wt.:wt.) to trace SIC-derived CO_2_ emissions, equivalent to 11.9 t lime ha^–1^ for a 10 cm topsoil layer (bulk density: 1.19 g cm^–3^). This mixture was repacked into 10 cm deep, chemically inert transparent plastics (2.5 × 10 × 10 cm) at 1 g cm^–3^ packing density, ensuring homogeneity and avoiding stratification that may dampen imaging quality (Figure 1). Initial soil moisture was set at 1.7% of the soil’s maximum water-holding capacity (WHC = 27% gravimetric water content) and increased to 60% using ultrapure/deionized water (EC = 18 udS) after liming.

**Figure 1:**
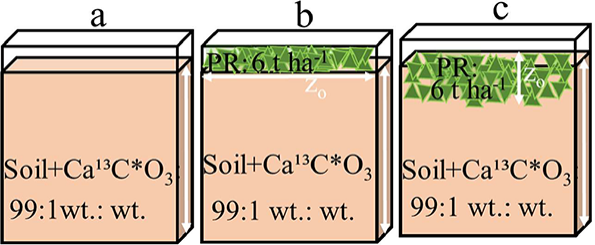
Two-dimensional schematic of the experimental setup showing (a) control (soil without residues), (b) mulching with plant residues (PR) placed on the surface, and (c) mixing with residues incorporated into ∼20% of the soil profile. All columns were incubated at 25 °C and 80% water saturation to assess how residue type (maize or wheat), quality (leaves or roots), and placement affect soil inorganic carbon (SIC) reactivity and CO_2_ fluxes. The column orientation enabled planar optode imaging.

Containers were then sealed within airtight rhizoboxes (12.5 × 12.5 × 5 cm), featuring a three-way valve for controlled gas exchange. Samples were preincubated at 25 °C for 7 days to stimulate microbial activation.

Maize and wheat residues (6 t ha^–1^) were applied, and moisture was raised to 80% WHC. Hydration was maintained throughout the 64-day incubation by monitoring hydrodynamics gravimetrically and replenishing any moisture deficit by adding water from the top every 7 days (Figure 2). This weekly interval provided moderate cyclic fluctuations (40–80% WHC) due to evaporation, representative of natural wet–dry cycles while avoiding excessive disturbance to gas diffusion and CO_2_ sampling. Maintaining this semistable moisture regime was essential to support microbial activity, facilitate carbonate reactivity, and ensure accurate δ^13^C-based CO_2_ source partitioning, thereby minimizing respiration impairment, pH alterations, and loss of isotopic resolution.

**Figure 2:**
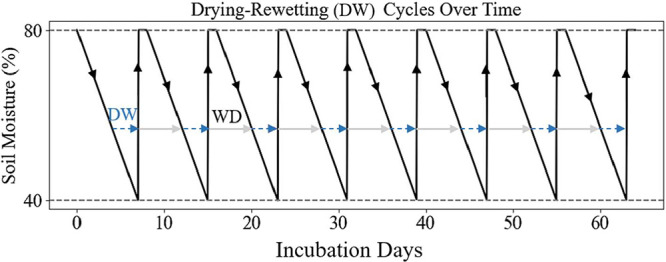
Sinusoidal pattern of drying–wetting (DW) and wetting–drying (WD) cycles during the incubation period, resaturated weekly to the optimum 80% moisture (determined by gravimetry). Alternating moisture fluctuations were induced by DW (blue dashed arrow) and WD (gray solid arrow) cycles.

Rhizoboxes were inclined at ∼45° throughout the incubation period to produce a uniform surface for optode imaging (PreSens GmbH, Regensburg, Germany). Treatments were ventilated passively to simulate a natural gas exchange.

The experiment evaluated how organic matter mineralization influences CaCO_3_ dissolution, CO_2_ emission, and acidification along a 10 cm soil profile under controlled conditions (Figure 1).

The use of different C sources having different isotopic compositions enabled us to differentiate their respective contributions to the total CO_2_ efflux from soil: The addition of ^13^C-labeled lime helped to differentiate between lime- and organic-C-derived CO_2_, while the use of maize (C4 plant; δ ^13^C = −13 to −14‰) vs wheat (C3 plant; δ^13^C = −29 to −28‰) roots and leaves enabled to ascertain organic-CO_2_ contribution. This resulted in a total of 9 treatments with 4 replications. Three factors simulated geochemical gradients typical of arable soils: (i) residue type (leaf vs root), (ii) application method (mixed into the topsoil at 0–2 cm vs surface-applied mulch), and (iii) limed soil without residues as a control. Treatments (Figure 1) were arranged in a completely randomized design with four replicates to analyze CO_2_ flux and pH shifts (ΔpH) due to respiration-induced microbial CO_2_ production, H^+^ release, and CaCO_3_ dissolution.

#### 2.1.2 Soil CO_2_ Emission Sampling

Gaseous samples were collected from each rhizobox at days 1, 3, and 8 and subsequently once every week until day 64. To ensure accurate measurements, the 531.3 cm^3^ headspace was flushed with CO_2_-free synthetic air (∼100 mL/min) for 30 min to flush out CO_2_ accumulated since the last gas sampling. Following flushing, the three-way valves were closed for 30 min to allow for CO_2_ accumulation. A 15 mL gas sample was then extracted via a syringe and transferred into an airtight and evacuated Exetainer (Labco Ltd.) vial for analysis. A second sample was taken after an additional 1 h of CO_2_ accumulation.

CO_2_ concentrations were measured using gas chromatography (7890B GC System, Agilent Technologies, Waldbronn, Germany), while δ^13^C isotopic composition of CO_2_ was analyzed via a GC (Trace Gas) coupled to an isotope ratio mass spectrometer (Isoprime 100, both Isoprime Ltd., Manchester, UK).^25^ Isotopic C-composition of solid samples was measured using an elemental analyzer (Isotope Cube) coupled to an isotope ratio mass spectrometer (Precision, both Elementar Analysensysteme GmbH, Langenselbold, Germany). CO_2_ flux was determined as the slope between CO_2_ amount in consecutive samples versus the chamber closure time. Spline interpolation was conducted to estimate fluxes on non-sampling days, and cumulative CO_2_ emissions were subsequently computed using Python.

### 2.2 Soil CO_2_ Efflux Partitioning

The contributions of soil inorganic carbon (SIC) and soil organic carbon (SOC) to CO_2_ efflux were quantified using isotopic partitioning:^26^

$$f_{\mathrm{SIC}}=\frac{\delta^{13}C_{{\mathrm{CO}}_{2}}-\delta^{13}C_{\mathrm{SOC}}}{\delta^{13}C_{\mathrm{SIC}}-\delta^{13}C_{\mathrm{SOC}}}$$ 1

where *f*_SIC_ represents the fraction of CO_2_ flux from calcite, δ^13^C_CO2_ is the isotopic signature of the emitted gas, δ^13^C_SIC_ corresponds to the calcite powder, and δ^13^C_soc_ denotes the soil organic carbon.

δ^13^C_soc_ was derived from the y-intercept of a regression between the two sampled values and their respective inverse time intervals. Because two organic carbon sources were present (native SOC and residue-derived C), only wheat residue treatments were used for isotopic partitioning. Wheat (a C_3_ plant) shares a similar δ^13^C signature with the native SOC (−26‰), allowing the system to be resolved using a two-source mixing model (SIC vs SOM). Maize residues (C_4_, δ^13^C ≈ −13‰) were excluded to avoid the need for a more complex three-source isotope mixing approach, which would introduce higher uncertainty given the temporal overlap of CO_2_ sources^26^ (Table 1).

**Table 1:** Properties of Soil and Plant Residues Used for the Incubation Experiment^a^

material	component	δ^13^C (‰)	C (%)	N (%)	C:N ratio	pH
soil		–26.04	0.54	0.09	6.3	7.2
maize residue	leaf	–13.18	0.36	0.02	17.5	
	root	–14.10	2.33	0.1	23.3	
wheat residue	leaf	–29.02	39.9	0.54	73.5	
	root	–27.99	45.8	0.54	85.4	

a C % and N % represent total carbon and total nitrogen, respectively.

To account for tracer variability in δ^13^C-enriched carbonate, flux partitioning calculations were based on the mean δ^13^C value of SIC (+102‰), incorporating its observed range (+82 to +122‰) for uncertainty analysis. Final SIC-derived CO_2_ fluxes were calculated by multiplying the fractional SIC–CO_2_ contribution by the total CO_2_ flux, with values interpolated and temporally integrated using Python-based spline interpolation and cumulative sum functions, as applied to total CO_2_ data. Since eq 1 provides integrated SIC–CO_2_ flux across the entire soil profile, depth-resolved carbonate turnover could not be directly derived using molar flux ratios. Instead, residual CaCO_3_ at discrete depths was quantified by acid-liberated CO_2_ following H_3_PO_4_ (80%) treatment at 40 °C,^14^ with sampling layers guided by optode-derived pH gradients. Temporal dynamics of CaCO_3_ dissolution, back-precipitation, and DIC redistribution were further computed through numerical modeling of the experimental data using HP1.^4,5^

### 2.3 pH Analysis

pH imaging was conducted on days 1, 16, 24, 32, 48, 56, and 64 using optode sensors (PreSens GmbH, Regensburg, Germany). These sensors detect photoluminescence changes in response to pH (Figure 3).^27^ A VisiSens TD camera captured fluorescence images with excitation at 470 nm and a DU02 detector for signal processing. Sensor foils (SF-HP5R) with a pH range of 5–8 were used, and data analysis was managed via VisiSens AnalytiCal 2 software.

**Figure 3:**
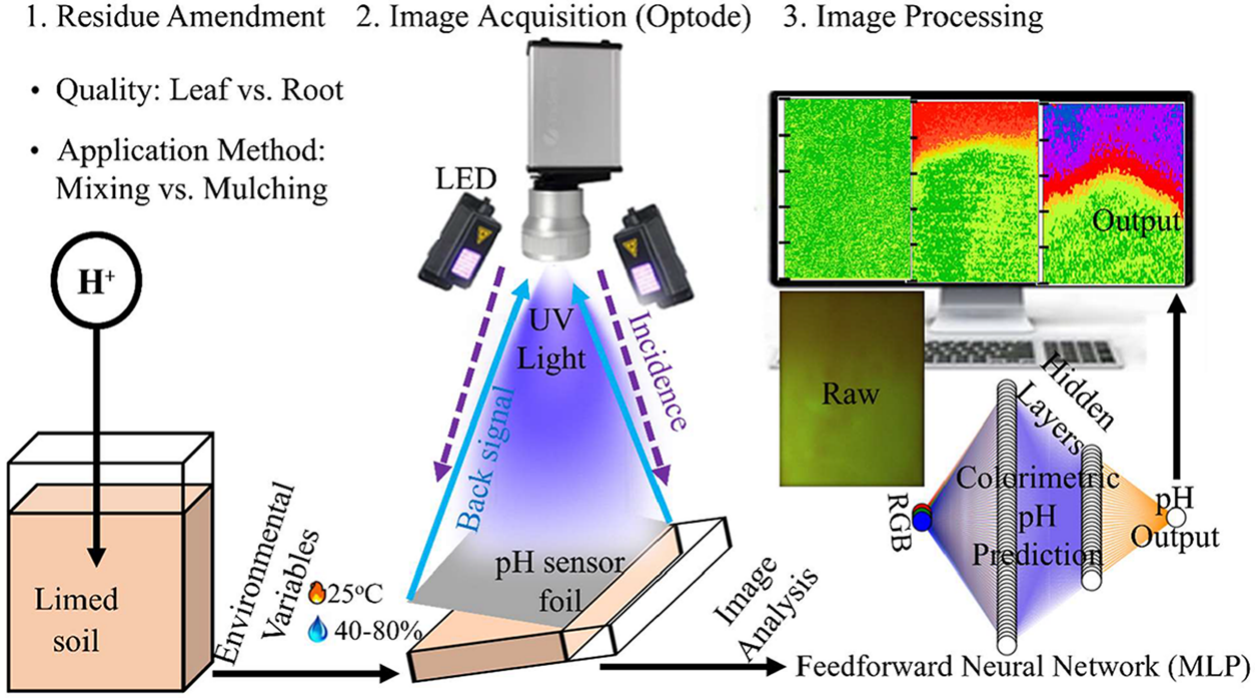
Experimental design and steps for visualizing spatiotemporal calcite sensitivity (in terms of pH changes) in soil profiles subjected to varying pCO_2_ elevation. (1) Limed soils (10 cm deep) in rhizoboxes were amended with two types of maize residues (leaf and root) and applied using two methods: mixing into the topsoil (0–2 cm) or surface mulching. Soils were incubated at 25 °C with drying–wetting cycles (40–80% soil–water saturation; see also Figure 2). (2) Optode imaging of the soil profile was conducted by opening the rhizobox from one side, attaching a pH sensor foil for reactive photoluminescence, and connecting the detector to a computer. Calibration images were captured using phosphate-buffered saline (PBS) solutions (pH 5.0–8.5) to establish a reference colorimetric function for pH quantification. (3) Multilayer perceptron (MLP) neural network was trained on calibration data and applied to process optode images, enabling high-resolution, *in situ* visualization of pH dynamics across soil profiles.

Each foil consisted of three layers: a polycarbonate support layer (125 μm), an analyte-sensitive layer (20 μm), and an optical isolation layer (20 μm). Foils (10 × 10 cm) were adhered to the transparent film and placed on the soil surface for 2 min. The camera, positioned 30 cm above at a 45° angle, captured fluorescence signals, with the green intensity decreasing as the pH lowered. Calibration images using phosphate-buffered saline (PBS) solutions (pH 5.0–8.5) enabled colorimetric pH mapping. Deep learning facilitated mapping of pixel-wise pH variations, using 62,500 pixels per image, with 90% for training and 10% for validation.^1^ All scripts (pHCODE) are available at P2KPTechSolutionsHub/OptoSense-AI.

After 64 days, soil was sectioned into five 2 cm layers. Air-dried samples were analyzed by using a pH glass electrode (WTW SenTix 81, Thermo Scientific) at a 1:1 water-to-soil ratio (Figure S1). The same layers were used for the δ^13^C analysis of residual calcite powder via acid combustion.

### 2.4 Experimental Constraints and Dissolution Modeling

To simulate the dissolution and potential recrystallization of labeled calcite (CaC*O_3_) under near-saturated flow conditions, we employed HP1, a coupled reactive transport modeling platform integrating HYDRUS-1D and PHREEQC.^2–6^ This framework captures interactions among water flow, solute transport, mineral equilibria, and carbonate speciation, enabling the dynamic assessment of HCO_3_^–^, Ca^2+^, and pH behavior during calcite reactivity under proton activity.

#### 2.4.1 Model Setup and Parameterization

The model domain consisted of a 10 cm silty loam soil column discretized into 101 nodes. Soil properties were defined by a bulk density of 1.2 g cm^–3^, a particle density of 2.65 g cm^–3^, and a porosity of 0.543 cm^3^ (Figure 4a). The initial soil solution was equilibrated with atmospheric CO_2_ fugacity (log_10_ *f*_CO2_ = −3.38, equivalent to 420 ppm, representing present-day conditions) and calcite to establish a representative baseline. Longitudinal dispersivity was set to 1 cm to account for solute dispersion (*D*_e_) within the porous medium (eq S5).^4,5^ The domain was homogenized with 1.2 × 10^–1^ mol kg^–1^ of calcite,^28^ reflecting the liming rate (Figure 1). Simulations were performed under steady-state, near-saturated conditions with a matric potential of −30 cm (≈80% water saturation). Water infiltration was set at 20 cm day^–1^, estimated via falling-head measurements.

**Figure 4:**
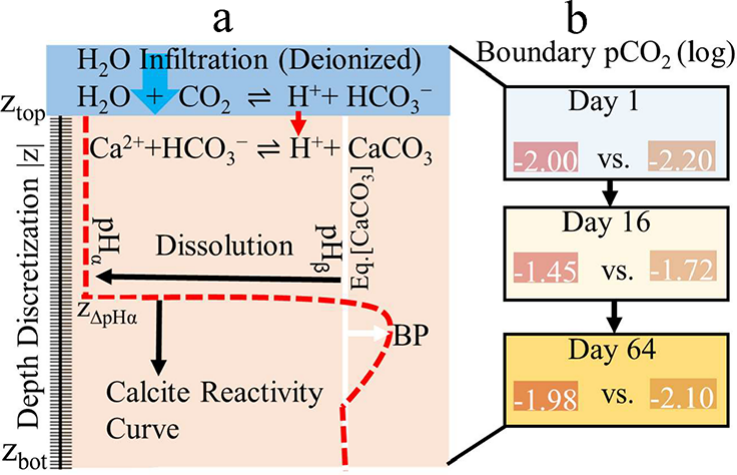
Summary of the model for calcite reactivity under transient biogenic CO_2_ inputs in a steady state near-saturated porous medium. Panel a: Infiltrating H_2_O (blue arrow) interacts with elevated pCO_2_ to form carbonic acid (H_2_CO_3_), which dissociates to release H^+^ and drive the dissolution of C*-labeled calcite. The system dynamically transitions between dissolution and back-precipitation as local equilibrium (white line) shifts (dashed curve) in response to depth-dependent pCO_2_–H^+^ elevation levered by time-variable boundary pCO_2_ (log scale). Panel b illustrates biologically mediated CO_2_ production under two residue treatments: mixed leaf litter (red box) and surface mulch (brown box). These evolving gradients regulate carbonate speciation and modulate calcite reactivity throughout the soil profile.

The Richards partial differential equation (eq S1) described the relationships between soil hydraulic functions, specifically water retention (θ­(*h*)) and hydraulic conductivity (*K*(*h*)). θ­(*h*) was defined by the closed-form equation of van Genuchten, while *K*(*h*)) followed the capillary model of Mualem (Table 2; see also eqs S2 and S3).^29,30^

**Table 2:** Hydraulic Properties of Silty Loam Soil Used for Solute Transport Modeling^a^

θ_s_ (cm^3^ cm ^–3^)	θ_r_ (cm^3^ cm ^–3^)	α (cm^–1^)	*n* (−)	*k*_s_ (cm day^–1^)
0.543	0.078	0.036	1.56	20

a θ_s_ and θ_r_ are the saturated and residual water content, respectively. *n*, *m*, and α are the empirical parameters, while *k*_s_ is the saturated hydraulic conductivity. *n*, α, and θ_r_ were calibrated using the silty loam pedotransfer function of Carsel and Parrish.^31^

#### 2.4.2 Geochemical Reactions and CO_2_ Boundary Conditions

The dissolution of C*-labeled calcite was driven by proton (H^+^) activity generated through hydrolysis of biogenic CO_2_ from microbial respiration according to the following reactions:

$${\mathrm{CO}}_{2}+H_{2}O⇌H_{2}{\mathrm{CO}}_{3}$$ 2

$$H_{2}{\mathrm{CO}}_{3}⇌{{\mathrm{HCO}}_{3}}^{-}+H^{+}$$ 3

$${{\mathrm{HCO}}_{3}}^{-}⇌{{\mathrm{CO}}_{3}}^{2-}+H^{+}$$ 4

$${\mathrm{CaC}}^{*}O_{3}+H^{+}\to {\mathrm{Ca}}^{2+}+{\mathrm{HC}}^{*}{O_{3}}^{-}$$ 5

CO_2_ partial pressures (pCO_2_) were varied at three time points (days 1, 16, and 64) to align with measured CO_2_ emissions. For the mixed leaf profile, time-variable log pCO_2_ values were set to −2, −1.45, and −1.98, while for the mulched profile, values of −2, −1.72, and −2.1 were applied (Figure 4b). These values reflect biologically induced CO_2_ accumulation under residue decomposition and regulate the rate of calcite dissolution (eq S11).^2,32^

To maintain the charge balance, the boundary solution was adjusted by modifying pH and carbonate speciation according to elevated CO_2_ conditions. The geochemical reactions governing calcite reactivity were defined in PHREEQC, ensuring dynamic interactions between solid-phase calcite and the aqueous phase. PHREEQC’s equilibrium phase module dynamically governed both forward dissolution and reverse precipitation of calcite, thereby mimicking buffering feedback at microsites (Figure 4a). This dual reactivity allowed tracking of net calcite transformations over time and depth. Simulations ran for 64 days using a maximum time step of 0.005 days for numerical stability. Outputs (pH, Ca^2+^, DIC speciation, and calcite saturation indices) were collected at 9 time points (days 1–64) across six depths (0, 1, 2, 3, 5, and 10 cm), providing high temporal and spatial resolution. GNUplot was used to visualize pH gradients, net mineral dissolution, and recrystallization fronts over time. This approach captures the dynamic calcite sensitivity under biological pCO_2_ elevations, advancing the mechanistic understanding of carbon turnover and mineral stability in porous media under elevated respiration regimes.

## 3 Results and Discussion

### 3.1 Amendment Type, Quality, and Application Method Modulate Total Soil CO_2_ Efflux

Soil CO_2_ efflux (μg C g^–1^ soil day^–1^) followed a distinct n-shaped temporal pattern across treatments (Figure 5a,b). The control, lacking organic inputs, consistently recorded the lowest efflux throughout the 64-day measurement period. In contrast, soils amended with maize residues produced higher CO_2_ emissions than those amended with wheat, and leaf amendments generally induced greater efflux than root amendments within each residue type. Application methods also played a significant role: mixed applications produced higher efflux than mulched ones.

**Figure 5:**
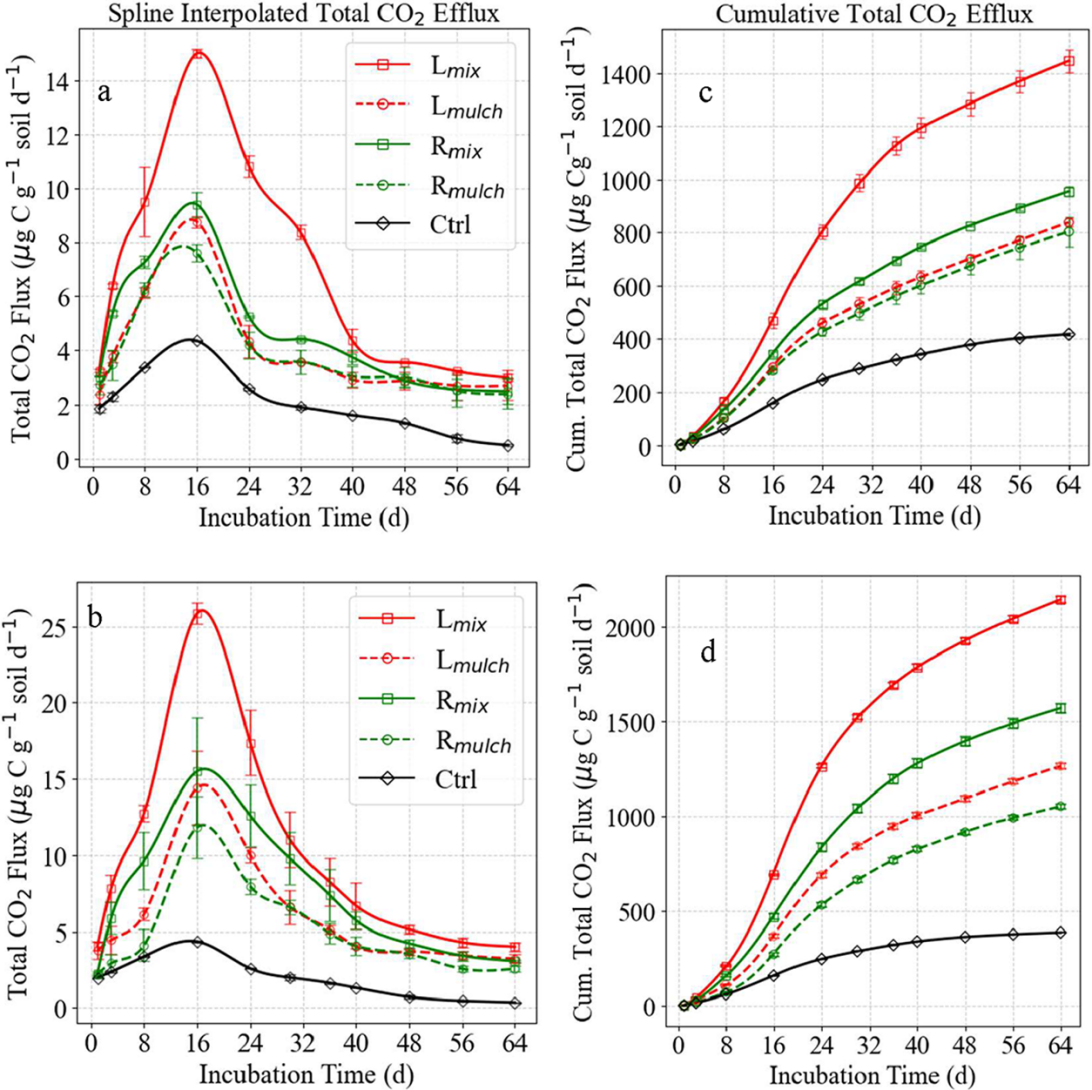
Soil CO_2_ efflux (μg C g^–1^ soil day^–1^) dynamics across residue amendments over 64 incubation days for wheat- (top) and maize- (bottom) amended soils, comparing leaf vs root residues and mix vs mulch applications. Efflux peaked on day 16, with *L*_mix_ treatments showing the highest flux due to greater microbial access to labile carbon. Maize residues, with lower C/N ratios, emitted more CO_2_ than wheat. Mulch applications sustained efflux longer, while the control remained lowest, reflecting carbon-limited microbial activity. Error bars show standard error (*n* = 4).

On day 1, mixed leaf treatments (*L*_mix_) yielded the highest initial flux (wheat: 3.24 ± 0.04; maize: 4.05 ± 0.14 μg C g^–1^ soil), while root-based treatments exhibited more gradual decomposition. CO_2_ efflux peaked on day 16, reaching 14.97 ± 0.11 μg C g^–1^ soil for wheat *L*_mix_ and 25.79 ± 0.34 μg C g^–1^ soil for maize *L*_mix_, both surpassing their respective root and mulch counterparts. Thereafter, emissions declined steadily and stabilized by day 64 (wheat *L*_mix_: 2.99 ± 0.07; maize *L*_mix_: 4.00 ± 0.15 μg of C g^–1^ soil).

Cumulative CO_2_ efflux increased sharply during the initial phase, followed by a more gradual rise between weeks 1 and 3, and plateaued toward the experiment’s end (Figure 5c,d). By Day 64, *L*_mix_ treatments showed the highest cumulative emissions (wheat: 1424.04 ± 11.14; maize: 2147.08 ± 16.16 μg C g^–1^ soil), while root-based and mulch treatments produced lower totals. The control consistently registered the lowest cumulative CO_2_ flux.

The observed differences in CO_2_ efflux across treatments can be attributed to the influence of residue type, biochemical quality, and application strategies on the decomposition kinetics. Maize residues, with lower C/N ratios (leaf: 17.3; root: 28.7) compared to wheat (leaf: 73.5; root: 85.0), supply a more nitrogen-rich and labile carbon source (Table 1). This facilitates faster microbial decomposition, thereby enhancing respiration rates and CO_2_ release.^33–35^ Leaf tissues, generally richer in soluble compounds, supported higher respiration rates, particularly under mixed applications that likely enhanced microbial access to the substrate.^36,37^

Mulch treatments moderated decomposition rates, likely due to limited residue–soil contact, resulting in more prolonged but lower emissions.^38,39^ In contrast, minimal CO_2_ efflux in the control reflects limited microbial activity due to carbon scarcity (Table 1).

These contrasting dynamics underscore how residue placement modulates the decomposition and carbon turnover. Optimizing residue management (through strategic selection of residue type and application methods) can therefore mitigate soil CO_2_ emissions, enhance carbon sequestration potential, and support long-term agroecosystem resilience.

### 3.2 Calcite Sensitivity to Elevated Biogenic CO_2_: Spatiotemporal Dynamics in Laboratory and Model Experiments

On day 0, all profiles followed uniform alkalinity (pH 7.8–8.0), indicating system saturation with calcite and the absence of acidifying activity (Figure 6a). By day 16, acidification gradients emerged, strongly modulated by residue quality and placement. The control maintained a stable alkaline regime (pH ≥ 7.8), underscoring minimal proton generation and robust buffering. Root mulch lowered pH by ∼1.0 unit to 6.7 at the surface (0–1 cm), with pH remaining near the baseline below 2 cm. Mixed root treatments intensified acidification to pH 6.5 in the 0–2 cm layer, indicating an enhanced interaction among residues, microbes, and carbonate minerals.

**Figure 6:**
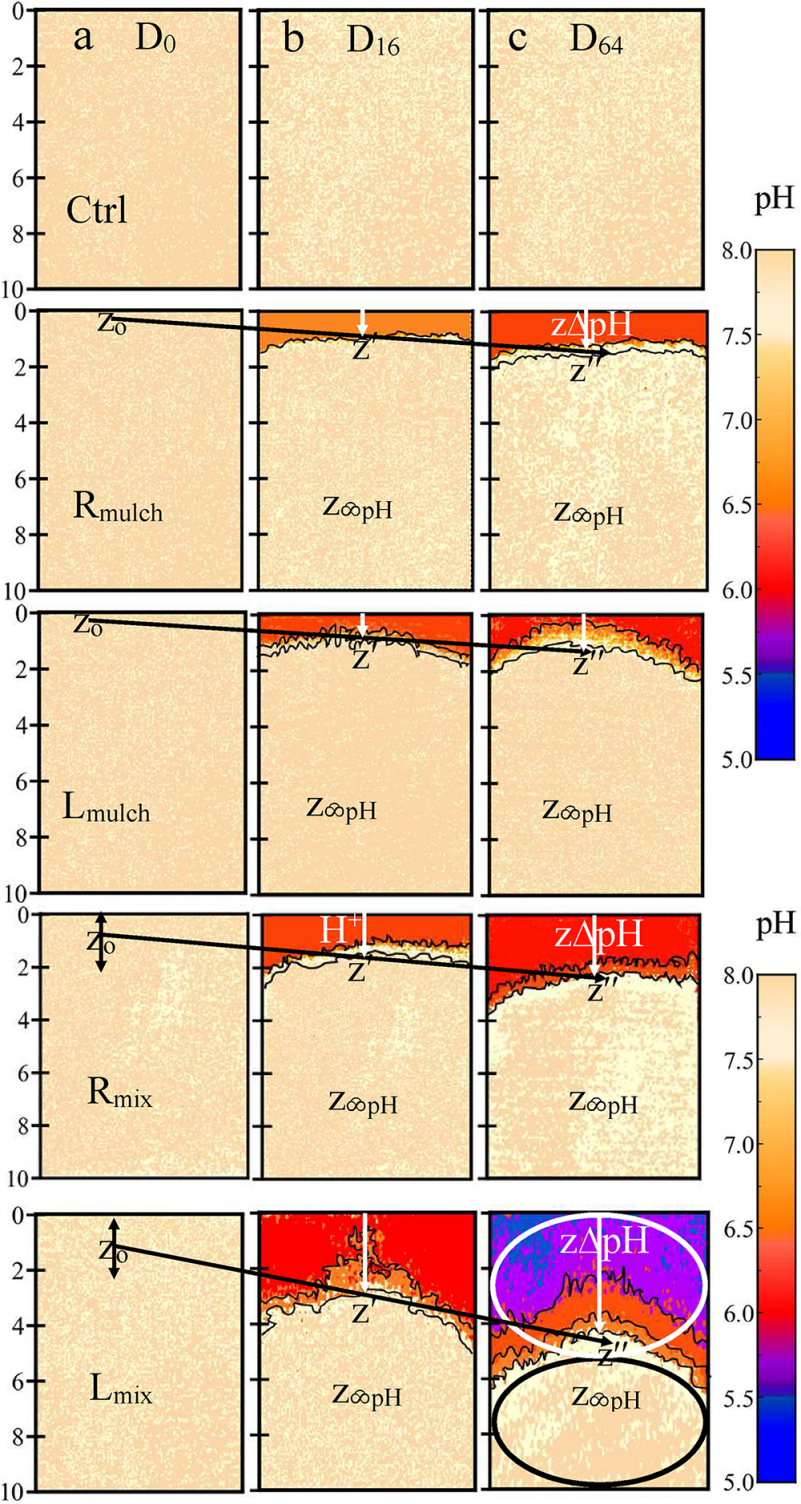
Average spatiotemporal pH gradient (inclined arrows) illustrating calcite sensitivity to elevated biogenic CO_2_ across residue types and placement strategies. High-resolution optode images show strong acidification in labile leaf and mixed treatments, with surface pH dropping to 5.7 and acidification fronts (*z*_o_, *z*′, and *z*″) extending beyond 5 cm by day 64. In contrast, root and mulched treatments caused milder acidification, while the control maintained a stable alkaline pH (7.8–7.9). Distinct pH stratification (scribbles) is evident across profiles, with *L*_mix_ inducing localized zones of extreme acidity (pH 5.5). The sensitive zone (*z*_ΔpH_, white oval) encroaches into the stable buffered zone (*z* 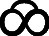
_pH_, black oval), driven by downward H^+^ transport (white arrow) from the residue applied zone (*z*_o_). These findings underscore calcite’s heightened reactivity to biogenic pCO_2_. Replicates and statistical significance are reported in Figure S2 and Table S3.

Leaf residues were more reactive. Mulched leaf residues caused a 1.6-unit drop to pH 6.2 at 0–1.5 cm (0.20 units day^–1^), while mixed leaf residues resulted in the sharpest decline—pH 5.8 at 0–2 cm and further to 6.2 and 7.0 in the 2–4 and 3–4 cm layers, respectively. Surface acidification rates reached 0.263 units day^–1^, exceeding root treatments by 3–4 times. These results reveal calcite’s heightened reactivity under elevated biogenic pCO_2_ generated by labile residue decomposition (Figure 6b).

By day 64, the acidification had deepened. The control remained buffered (pH ∼ 7.8). In root mulch, pH dropped to 6.5 (0–1 cm) and 6.4 (1–2 cm), while mixed root treatments reached pH 6.0 (0–2 cm), with circumneutral pH recovered below 3–4 cm. Leaf mulch sustained acidified conditions (pH 6.0–6.7 in the upper 3 cm), whereas the mixed leaf treatment exceeded calcite tolerance thresholds (surface pH declined to 5.5) and fronts extended to 5.2 cm. The 2–4 cm zone recorded the steepest pH gradient (∼0.023 units day^–1^, 10-fold higher than adjacent layers), indicating sustained proton pressure and carbonate depletion.

Numerical simulations reproduced the laboratory results, capturing the spatiotemporal pH dynamics observed by optode imaging (Figure 7a,b). Calcite dissolution patterns further supported these findings. Intact calcite at the simulation start dissolved rapidly in mixed profiles, with complete depletion within ≈5.2 cm (52% of column depth) by day 64 under elevated CO_2_-induced proton attack (Figure 7c,e). In contrast, mulched profiles showed slower dissolution confined to the upper 19% of the column (Figure 7d,f). Downward transport of dissolution products (Ca^2+^, HCO_3_^–^) triggered local supersaturation and minor back-precipitation (ΔCaCO_3_ > 0) in sublayers (Figure 7e,f, green arrows). This back-precipitation was attributed not to *in situ* dissolution but to Ca^2+^ redistribution with percolating water, reflecting coupled acidification–neutralization dynamics.

**Figure 7:**
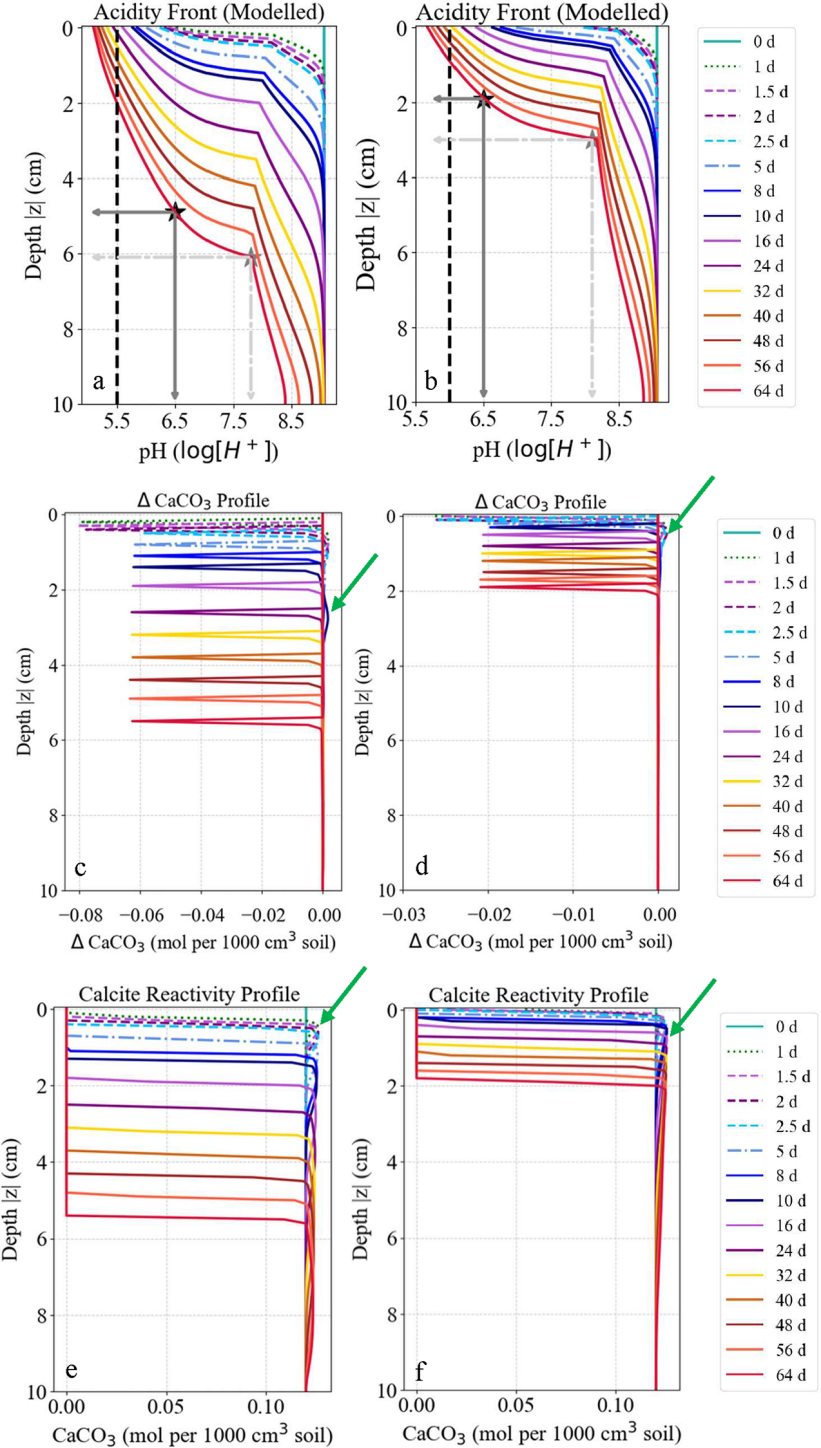
Modeled calcite reactivity under biogenic pCO_2_ elevation. Initial alkaline, calcite-buffered pH (∼8.5) gradually decreases over time, reaching 5.5 (mixed; a) and 6.2 (mulched; b) by day 16, with the acidity front extending deeper in mixed treatments (black dashed line). Negative calcite changes reflect buffering capacity exceedance and indicate greater depletion in mixed residues (0–5.7 cm; c) than mulched (0–2 cm; d) by day 64. Calcite dissolution, initiated earlier and progressing deeper in mixed (e) than mulch amendments (f). The boundaries of carbonate-depleted and carbonate-containing depths are shown by black and gray stars and the corresponding lines in (a) and (b). Localized calcite recrystallization (e and f, green arrows) in underlying layers, driven by downward transport of Ca^2+^ and HCO_3_^–^ from upper-layer dissolution, indicates secondary accrual at supersaturated carbonate–aqueous interfaces.

The pH decline observed in residue-amended soils is attributable to enhanced microbial respiration, which generates CO_2_ and organic acids.^35–37^ Decomposition byproducts, through carbonic acid formation and proton release, drive acidification and interact with calcite, accelerating its dissolution.^40^ The extent of acidification was modulated by residue quality and the application method. Root residues, characterized by a higher C:N ratio (28.7), decomposed more slowly and produced more localized acidification at the surface. In contrast, lower C:N ratios (17.3) of leaf residues enhanced rapid decomposition, resulting in greater CO_2_ production and deeper acidification fronts.^35,39^

The stronger acidification in mixed applications can be attributed to increased soil–residue contact, which facilitates microbial accessibility, colonization, and metabolic activity.^38,41^ Mulched applications exhibited more surface-restricted acidification, likely due to limited microbial access and greater physical separation from active microbial zones.

The buffering role of calcite was evident but was transiently overwhelmed under biogenic pCO_2_ elevation, particularly in mixed leaf treatments. Buffering effects were more pronounced in deeper layers (*z* = 6–10 cm, 2–10 cm), whereas upper horizons (*z* ≤ 6, 2 cm) experienced acute acidification due to downward percolation of CO_2_-rich byproducts (Figures 6 and 7).

The fine particle size of the applied calcite likely increased its surface area and reactivity, supporting rapid initial buffering.^4,35^ However, persistent acidification due to elevated pCO_2_ led to substantial CaCO_3_ dissolution, especially in the topsoil.^39,40^ Once the acidification threshold was exceeded, the buffering capacity collapsed rapidly in the upper soil layers, while deeper layers remained protected by carbonate saturation, unless ion transport induced secondary precipitation or further dissolution.^2–6^ The coupling of forward dissolution and spatiotemporally lagged recrystallization illustrates the dynamic nature of calcite as both a reactive buffer and a mobile mineral phase, driven by water transport and pCO_2_ biogeochemical gradients.^28^

Model simulations effectively captured these pH dynamics, including the timing and depth of acidification fronts and calcite depletion, reflecting the laboratory representativeness of the parametrized time-variable pCO_2_ inputs (Figure 7). The strong agreement between modeled and measured values confirms the model’s utility in projecting carbonate buffering responses under organic amendment-induced acidification scenarios.

These findings reinforce the importance of understanding the interplay among biogenic CO_2_, organic amendment quality, and application strategy when evaluating carbonate stability in soils. The conditional buffering capacity of CaCO_3_ under microbial acidification pressures must be critically considered in soil management strategies aiming at long-term carbon retention, acid neutralization, and pH stability in agroecosystems.

### 3.3 Mechanisms Driving Calcite Reactivity: Implications of Particulate Nature of Soil Solution on Earth System Processes

#### 3.3.1 Depth-Resolved Acidification, Calcite Dissolution Fronts, and Drivers

Depth-resolved calcite reactivity revealed intensified acidification in mixed residue-amended profiles, with the pH decreasing to 5.5 at 0–2 cm, 5.8 at 3 cm, and 6.56 at 5 cm by day 64 (Figure 8a). This contrasts with mulched profiles, where surface pH remained higher (5.8 at 0 cm, 6.05 at 1 cm, and 6.56 at 2 cm) and deeper layers retained their alkalinity (pH ≈ 9.1; Figure 8b). These acidification trends aligned with calcite depletion patterns: in mixed profiles, H^+^ attack rapidly consumed 66.67% of reactive calcite within the first 3 days (0.08 mol/day), dropping to 41.67% by day 24, and leading to complete dissolution within 0–5 cm by day 64 (Figure 8c). Mulched profiles showed slower and incomplete depletion (∼23.33% initially, stabilizing at 18.33% by day 32), with residual calcite persisting at a 2 cm depth (Figure 8d).

**Figure 8:**
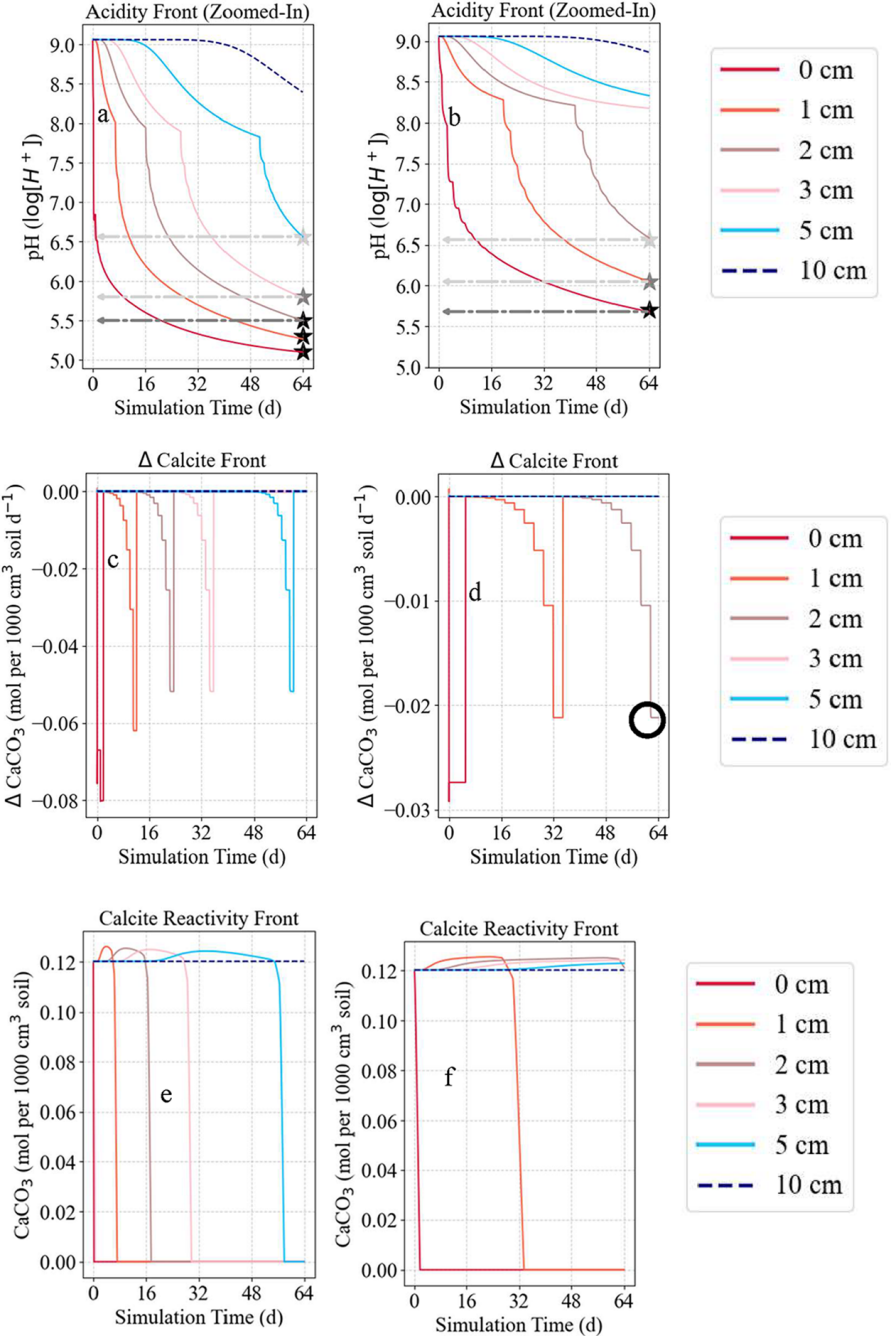
Zoomed-in depth-wise (|*z*| = 0, 1, 2, 3, 5, and 10 cm) variation in pH in mixed (a) and mulched (b) profiles, showing more rapid acidification and deeper H^+^ export in mixed profiles with steepest gradients occurring when more than 60% of the CaCO_3_ is depleted. Calcite depletion rates in mixed (c) and mulched (d) profiles with higher initial dissolution rates in mixed profiles. Temporal progression of calcite dissolution in mixed (e) and mulched (f) profiles, showing complete depletion at 0–5 cm in mixed profiles by day 64 and slower dissolution in the mulched profiles. These trends highlight residue-driven acidification, microbial respiration, and porewater chemistry as key regulators of soil carbonate dynamics. Circles represent pCO_2_ and time-constrained incomplete H^+^ attack (d) and dissolution (f) events. Stars (a, b) represent acidity front propagation.

These patterns reflect greater biogenic CO_2_ accumulation and concomitant protonation and proton transport in mixed residue-amended profiles. High initial pH from liming (pH 9.1) enhanced microbial respiration, increasing pCO_2_ and driving a positive feedback loop where CO_2_ dissolution and organic acid release exceeded calcite buffering capacity.^36,42^ In mulched profiles, constrained proton generation and influx delayed and attenuated dissolution, retaining 8.33% calcite at 1–2 cm (Figure 8f). Residue quality further influenced the reactivity. Mixed leaf residues with low C:N ratios accelerated microbial activity and H^+^ release compared to root residues (Table S2).

Moreover, water infiltration and pore water chemistry modulate the ionic strength and buffering.

Although ultrapure irrigation water (EC < 18 μS cm^–1^) minimized external acid loading, it facilitated *in situ* H^+^ redistribution through convective transport (Figure 2), mimicking processes in natural systems where organic acids leach from topsoil to subsoil.^43,44^

These findings reflect the likely outcomes under climate-driven acidification. Acid rain, subsurface CO_2_ injection, and reduced gas diffusivity increase pCO_2_ and enhance calcite solubility,^11–13^ particularly in the Global South (e.g., rapidly industrializing regions in Asia) where SO_2_, NO_*x*_, and CO emissions are projected to rise.^24,45,46^ Even under more saturated conditions (>80% WFPS), hydraulic fluxes amplify proton transport, inducing preferential flow that intensify acid front migration to deeper depths where remediation remains costlier,^4^ highlighting the importance of evaluating topsoil lime-buffering efficacy on short temporal (diurnal to monthly) scales, as high microbial turnover can deplete carbonate reserves rapidly, a factor often overlooked in no-until systems.

#### 3.3.2 System-Level Implications: pCO_2_-Mediated Dissolution, SIC Fluxes, and Environmental Feedbacks

Calcite reactivity is governed by both acid–base equilibria and molecular-scale interactions, including water adsorption on surface Ca sites.^2^ Unlike consolidated limestone, particulate calcite is more susceptible to H^+^ attack due to its high surface-area-to-volume ratio and hydration sensitivity (Figure 6). High clay content (25%) enhances Ca^2+^ sorption and hydration on negatively charged surfaces, weakening Ca–O bonds and accelerating dissolution under acidic conditions (Figure 6).^47^

Respiration-induced H_2_CO_3_ formation and dissociation contribute additional H^+^, exacerbating alkalinity loss and mobilizing Ca^2+^ and bicarbonate into deeper horizons (Figure 7). Under near-saturated flow conditions (matric potential = −30 cm), convective transport dominates, with effective dispersion (*D*_e_) exceeding diffusion by over 30-fold.^3,48^ This hydrodynamic regime enables rapid acid front propagation, as confirmed by HP1 simulations (Figures 5–8). Rewetting further enhances H^+^ translocation and SIC depletion, intensifying the topsoil decarbonation.

Soil physical and chemical properties modulate these processes. High silt and clay fractions improve ion retention and facilitate metal sorption (Ca, Mg, K), strengthening interactions with organic ligands (Figure S1a).^48^ At moderate acidification (pH 5–7), improved nutrient bioavailability and water quality can boost soil fertility and ecosystem resilience.^38^ However, below pH 5, emerging Al^3+^ and H^+^ toxicities compromise soil health and ecosystem functions.^36^

Cumulatively, dissolution resulted in substantial SIC losses. Mixed profiles exported 0.836 t C ha^–1^ (0–5 cm), with peak fluxes near the surface (0.0202 g C kg^–1^ soil), tapering with depth (Figure 9d; Table S4). In contrast, mulched profiles lost only 0.0552 t of C ha^–1^ (0–1 cm; Figure 9c). These losses exceeded intrinsic buffering capacity by up to 99.9% in the surface layers of mixed profiles (*z* = 0–5 cm) and 96.4% in mulched ones (*z* = 0–1 cm) (Figure 9a,b), confirming microsite carbonate disequilibrium and decarbonation.

**Figure 9:**
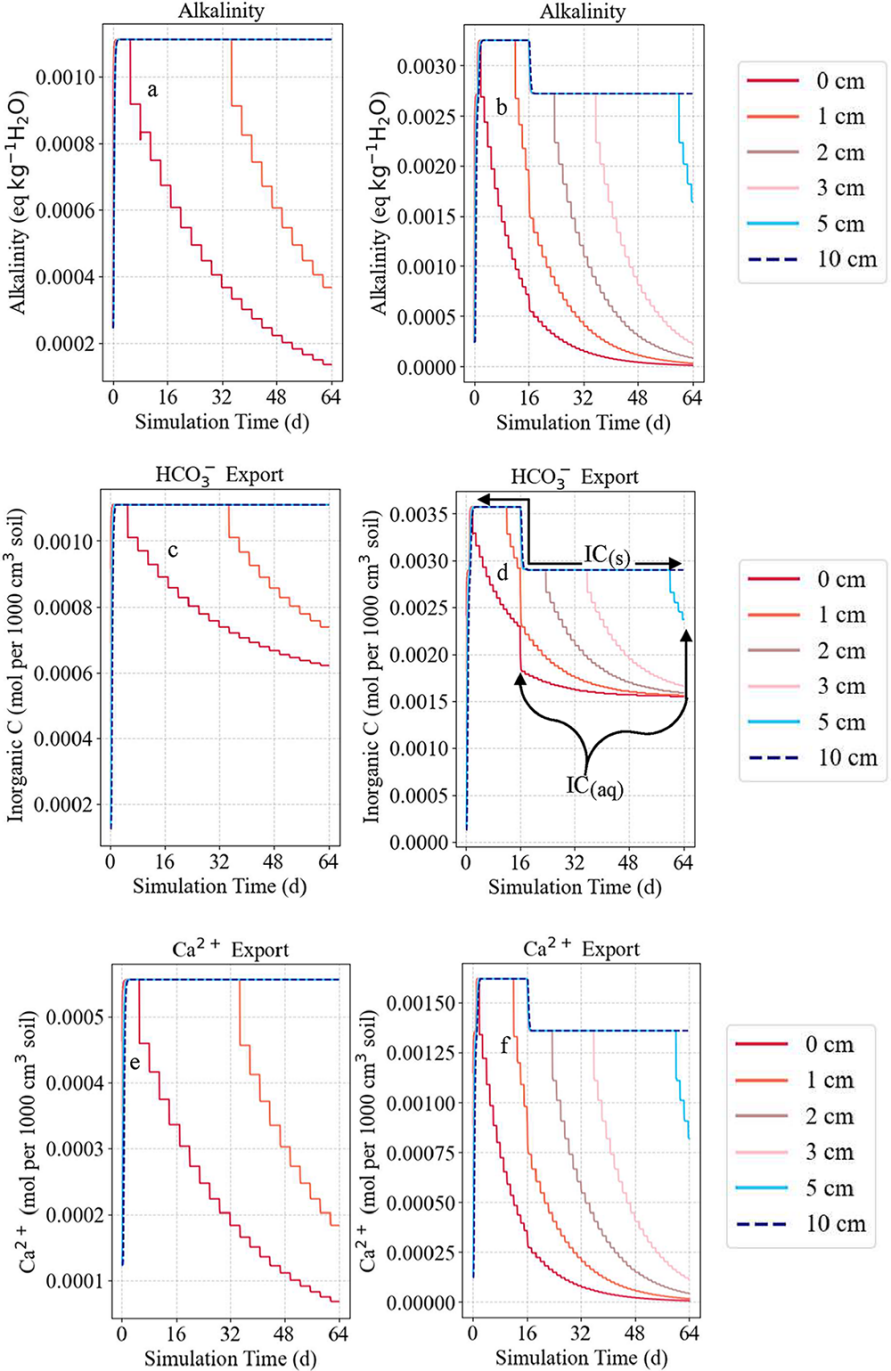
Disruption of soil solution below equilibrium alkalinity (left) and enhanced bicarbonate export (top) in response to elevated pCO_2_ from organic residue decomposition, reinforcing soil respiration rates as a key influencer of the acid-carrying capacity of soil pore waters in mulch-amended (left) and mixed-leaf (right) profiles. (a, b) Alkalinity buffering exceedance across depths. (c, d) Depth-resolved SIC export and bicarbonate fluxes. (e, f) Free Ca^2+^ fluxes with depth following calcite dissolution. Electrical balance, ionic strength, and DIC stocks are shown in Figure S3 and Tables S4–S5.

Such bicarbonate fluxes facilitate vertical carbon transfer from the vadose to phreatic zones and ultimately into fluvial systems, reshaping terrestrial–aquatic carbon coupling.^32^ This vadose-to-phreatic zone bicarbonate migration highlights soil respiration rates as a key influencer of the acid-carrying capacity of pore waters (Figure 9a), directly shaping carbonate transformations and subsurface alkalinity dynamics.^4,22,49^

Beyond carbonate dynamics, declining alkalinity and buffer capacity increase the mobility of agrochemical contaminants (such as pesticides and fertilizers) by weakening ligand–clay complexes and enhancing solubility.^50,51^ These risks are magnified under high moisture and macropore flow, which disaggregate clay–organic floccules and expedite contaminant transport to groundwater.^3,47^

HP1 simulations integrate thermodynamic and kinetic processes, offering a mechanistic framework to model dissolution fronts and carbonate reactivity under variable pCO_2_ conditions, crucial for anticipating soil buffering breakdowns in response to changing climate and land use.

Simulated calcite dissolution has significant implications for soil CO_2_ efflux and the global C cycle across immediate, short-, and long-term scales. Elevated biogenic CO_2_ from SOC mineralization and residue decomposition is temporarily mitigated by δ^13^C*-labeled calcite (CaC* O_3_, eq 5), with released HC*O_3_^^–^^ remaining dynamic and responsive to changes in soil chemistry and hydrodynamics. Under (near) saturated soil water, HC*O_3_^^–^^ can be leached to deeper soil layers, leading to the decarbonation of the topsoil and deep C* sink (Figure 9b,d), potentially sequestering C over geological time scales.^52^ Under acidic conditions, HC*O_3_^^–^^ reactivity with protons (H^+^) may lead to C*O_2_ formation (eq 6), while under alkaline conditions, HC*O_3_^–^ recrystallizes into CaC*O_3_, also liberating C*O_2_ (eq 7)­

$${\mathrm{HC}}^{*}{O_{3}}^{-}+H^{+}⇌C^{*}O_{2}+H_{2}O$$ 6

$${\mathrm{Ca}}^{2+}+2{\mathrm{HC}}^{*}{O_{3}}^{-}⇌{\mathrm{CaC}}^{*}O_{3}+C^{*}O_{2}+H_{2}O$$ 7

This equilibrium illustrates that for each mole of dissolved CaCO_3_, one mole of carbon can be transiently sequestered. However, the reaction is reversible; under conditions of reduced pCO_2_, due to degassing, drying, or elevated temperatures, calcite can recrystallize, negating its net contribution to CO_2_ emissions. This carbonate sensitivity underscores the need to prioritize organic amendments over inorganic ones, as the latter can supply protons that promote net CaCO_3_–CO_2_ release.^53^

Integrating these mechanistic insights enhances our understanding of soil carbon transformations and the stability of calcite under varying environmental conditions. This is essential for accurately assessing carbon fluxes and developing effective deep-soil carbon sequestration strategies in the context of climate change mitigation.

### 3.4 Mixed Continuum Nature of Soil CO_2_ Emission: Looking into the Black Box

The δ^13^C values of the efflux of CO_2_ from residue-amended soils revealed clear temporal shifts in the relative contributions of soil inorganic carbon (SIC) and soil organic matter (SOM) to total CO_2_ emissions (Figure 10a). On day 1, δ^13^C values in residue-treated soils were notably enriched (e.g., *L*_mix_: +25.92‰; *L*_mulch_: +25.11‰), while the control showed a slightly higher value (+29.22‰). These initial values reflect background SIC-derived CO_2_ equivalent to ∼7.5 × 10^–6^ mol CaCO_3_ per kg soil (approximately 0.0063% of applied calcite), following dissolution in water.

**Figure 10:**
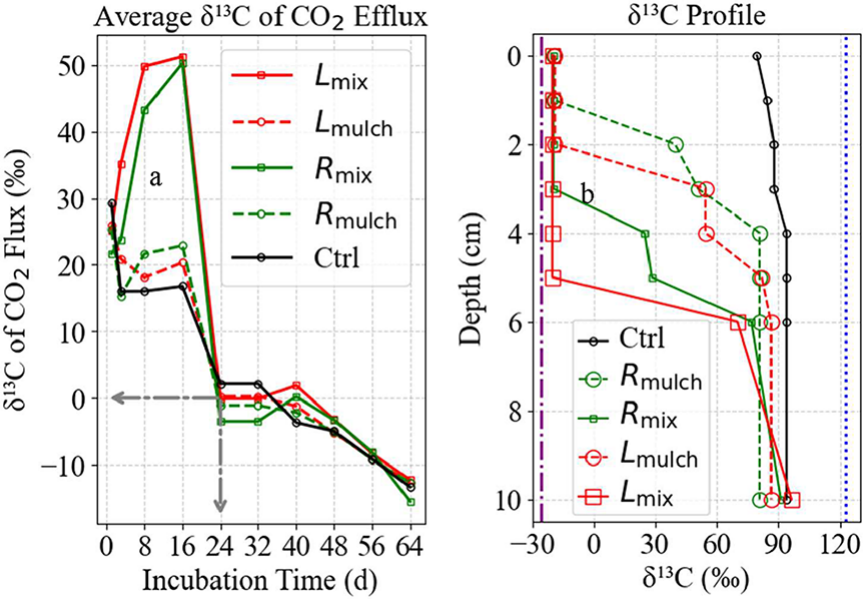
δ^13^C dynamics of soil CO_2_ efflux and SIC stability: (a) Temporal evolution of δ^13^C values in soil CO_2_ efflux, illustrating the transition from SIC-derived to SOM-derived CO_2_ over 64 days. Early enrichment in δ^13^C (days 1–16) indicates dominant calcite dissolution, while declining values after day 24 mark a shift toward SOM-controlled respiration. (b) Postexperiment δ^13^C profiles across soil depths (0–10 cm), highlighting SIC stability in deeper layers (6–10 cm) and enhanced calcite depletion in surface layers (0–5 cm) due to elevated pCO_2_ from microbial respiration in residue-amended treatments. The gray lines on (a) mark the transition at day 24, where SIC’s contribution was ∼20% (Figure 11e, horizontal arrow), corresponding to δ^13^C values of ∼0‰. Purple and blue dashed-dotted lines on b represent background δ^13^C of SOM and enriched CaCO_3_–C.

By day 8, δ^13^C values increased markedly, peaking in *L*_mix_ (+49.79‰) and *R*_mix_ (+43.27‰), with more moderate increases in mulched treatments. This trend intensified by day 16, reaching maximum δ^13^C values in *L*_mix_ (+51.29‰) and *R*_mix_ (+50.27‰), indicating a dominant contribution of δ^13^C-enriched CO_2_ from calcite (initial δ^13^C = +122‰; Figure 8b). These peaks coincide with enhanced microbial activity, elevated soil pCO_2_, and acid production, conditions that accelerate calcite dissolution.^34,54,55^

A sharp inflection occurred by day 24, with δ^13^C values in *L*_mix_ dropping to near-neutral (−0.10‰), suggesting a balanced contribution from SIC and SOM. By day 64, all treatments exhibited negative δ^13^C values (*L*_mix_: −12.27‰; *R*_mix_: −15.52‰), indicating the dominance of SOM-derived respiration. This transition from SIC to SOM dominance illustrates a temporal continuum of CO_2_ sources driven by microbial decomposition and changes in calcareous soil chemistry.

Vertical δ^13^C profiles at the experiment end reinforced these dynamics (Figure 10b). In the control, δ^13^C values remained stable around +92‰ throughout the 0–10 cm profile. In contrast, amended treatments showed significant δ^13^C reductions in surface layers: 0–5.2 cm (*L*_mix_), 0–3 cm (*R*_mix_), and 0–2 cm (*L*_mulch_). These isotopic depletions were strongest in mixed leaf treatments, likely due to enhanced microbial activity and CO_2_ production. Subsoil δ^13^C values (+89–92‰) remained relatively constant, indicating minimal calcite alteration at depth, possibly buffered by upper soil layers.^2,40^

The spatial and temporal δ^13^C patterns affirm a mixed continuum model of soil CO_2_ emission, with SIC and SOM contributions varying as functions of residue quality, microbial activity, and placement method. Residue mixing notably intensified the SIC phase, prolonged the δ^13^C peak, and deepened the isotopic shift—suggesting higher CO_2_ flux and calcite reactivity compared to surface mulching.

These findings expand upon traditional two-endmember mixing frameworks,^26^ by revealing more nuanced transitions in CO_2_ source dynamics over time and depth. The δ^13^C inflection at ∼0‰ (Figure 11e) marks a critical threshold where SIC and SOM contributions become approximately equal—a valuable indicator for mechanistic modeling.

**Figure 11:**
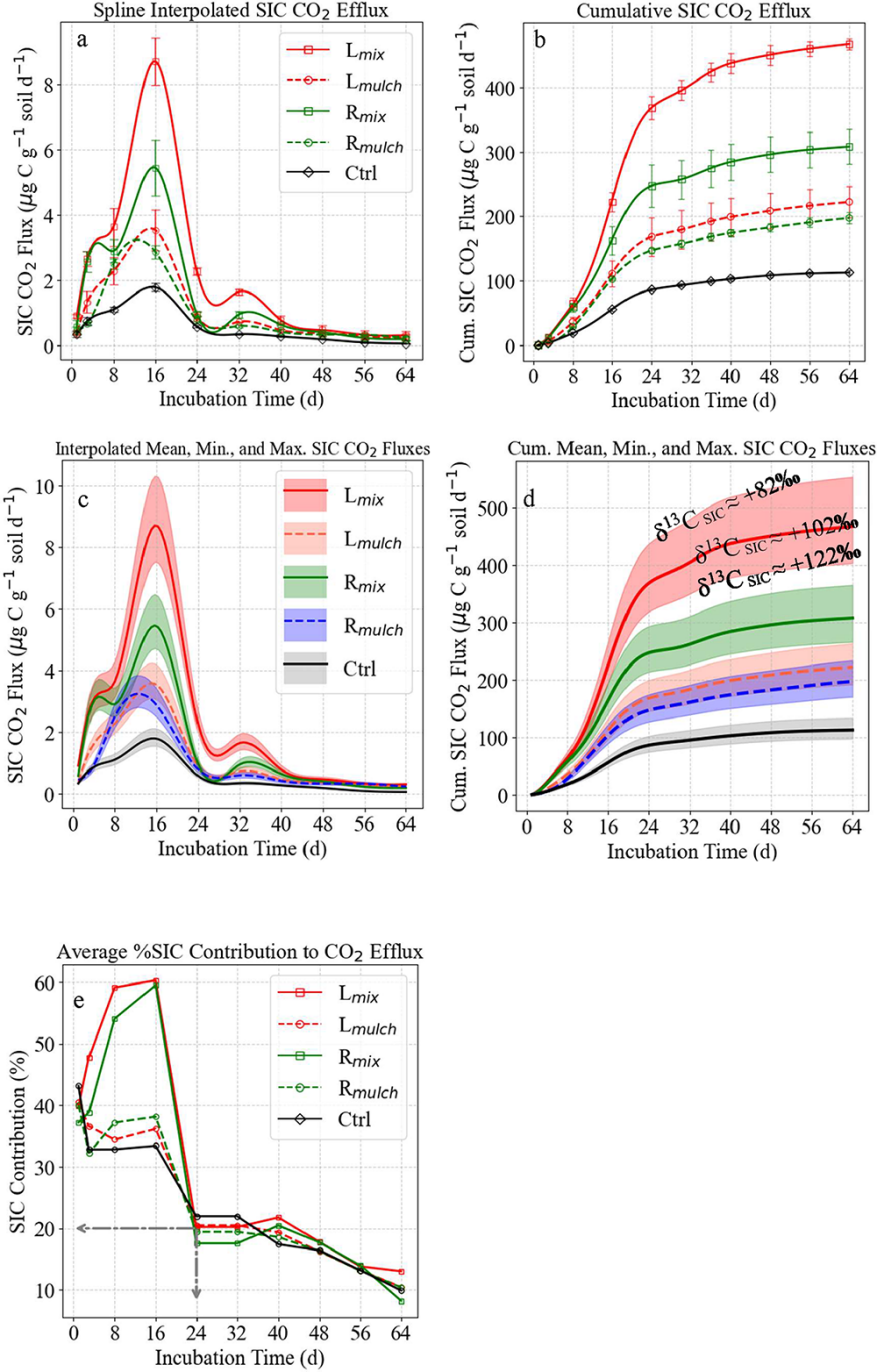
(a) Temporal evolution of calcite-derived CO_2_ efflux across treatments, showing peak SIC contribution at day 16, followed by a decline as calcite dissolution rates slowed. (b) Cumulative CO_2_ efflux highlighting the dominant role of mixed wheat leaf residues in accelerating calcite decomposition and SIC release. Whiskers on a and b are standard errors (*n* = 4). (c) SIC-derived CO_2_ efflux and (d) cumulative SIC contribution based on mean, highest, and lowest δ^13^C values of the applied calcite powder, illustrating the variability in isotopic contributions and its impact on total CO_2_ emissions. The shaded areas on c and d are to consider the measured min and max. δ^13^C values of the labeled CaCO_3_ mixture. (e) Percentage contribution of SIC-derived CO_2_ over time, demonstrating its initial dominance before transitioning to soil organic matter (SOM)-driven respiration by day 64.

However, δ^13^C-based estimates may overstate SIC-derived CO_2_ contributions, especially where recrystallization or CaCO_3_ reprecipitation occurs in deeper profiles. Such processes, invisible to surface measurements, necessitate caution in interpreting δ^13^C signals.^56^ Hence, future assessments should integrate isotopic tracing with geochemical modeling to account for transport, buffering, and re-equilibration phenomena. The enhanced reactivity in mixed treatments underscores the importance of residue quality and placement in managing SIC stocks and mitigating unintended CO_2_ emissions. High-resolution isotopic monitoring, coupled with reactive transport modeling, is essential for advancing the predictive understanding of carbon cycling in calcareous, residue-amended systems.

### 3.5 Contribution of SIC to CO_2_ Efflux: Implications for Global Carbon Accounting

The decomposition of δ^13^C-labeled calcite was monitored over 64 days to quantify the contribution of soil inorganic carbon (SIC) to total CO_2_ efflux relative to that derived from soil organic matter (SOM). On day 1, calcite-derived CO_2_ efflux was low but detectable, highest in *L*_mix_ (0.90 ± 0.03 μg C g^–1^ soil day^–1^), followed by *R*_mix_ (0.57 ± 0.12), with mulch applications and the control emitting less (Figure 11a). The percentage contribution of calcite-derived CO_2_ was already substantial in *L*_mix_ (39.90%) and *L*_mulch_ (40.53%), while the control exhibited a slightly higher proportion (43.11%), likely reflecting background SIC dissolution (Figure 11e).

By day 8, the level of CO_2_ efflux increased sharply across treatments. *L*_mix_ recorded the highest calcite-derived efflux (3.67 ± 0.34), followed by *R*_mix_ (2.90 ± 0.20) and the control (1.10 ± 0.05). The proportion of SIC-derived CO_2_ rose significantly in mixed treatments (*L*_mix_: 59.14%; *R*_mix_: 54.06%) compared to mulched applications (*L*_mulch_: 34.42%; *R*_mulch_: 37.20%).

Peak CO_2_ efflux occurred on day 16, with *L*_mix_ reaching 8.70 ± 0.43 μg C g^–1^ soil day^–1^ and *R*_mix_ reaching 5.45 ± 0.49. Percentage contributions of SIC peaked at 60.31% in *L*_mix_ and 59.52% in *R*_mix_, indicating intense carbonate dissolution (Figure 11e). This rapid increase reflects microbial stimulation and associated acidification, enhancing the calcite solubility.

From day 32 onward, calcite-derived CO_2_ efflux declined across all treatments. Though *L*_mix_ remained the highest emitter (1.63 ± 0.06), its SIC-derived proportion dropped to 20.23%, with further declines to 13.03% by day 64. *R*_mix_ and the control followed a similar trajectory, ending at 8.21 and 5.34%, respectively. These results suggest that the primary phase of calcite weathering concluded by day 32, with residual emissions persisting under declining microbial activity and reduced acid input.

Cumulative SIC-derived CO_2_ emissions were consistently the highest in *L*_mix_ across all time points (Figure 11b). By day 16, *L*_mix_ had released 222.33 ± 14.65 μg of C g^–1^ of soil, 4-fold higher than the control. By day 64, totals reached 467.02 ± 8.48 (*L*_mix_), 307.95 ± 26.75 (*R*_mix_), 222.38 ± 24.10 (*L*_mulch_), and 112.95 ± 3.35 (control), confirming that residue placement and quality strongly modulate SIC responses.

These findings align with Ramnarine et al.,^26^ who observed significant SIC-derived CO_2_ (∼74%) during early decomposition under elevated microbial activity. Likewise, Tamir et al. reported ∼30% SIC-derived fluxes under controlled lab conditions,^57^ mirroring our observations of a transient peak in SIC emissions.

The initial low SIC efflux, followed by a sharp increase, suggests a lag phase during which microbial communities acclimate to residue inputs. Enhanced microbial respiration likely lowered local pH, accelerating calcite dissolution, especially in *L*_mix_ treatments, where residue–soil contact, lability, and bioavailability were optimized. The dominance of SIC-derived CO_2_ until day 16 and its subsequent decline mirror the progressive exhaustion of labile organic compounds, reinforcing a coupled mineral–organic carbon turnover dynamic.^26,57^

The sustained yet declining SIC emissions post day 32 demonstrate the long-tail effect of residue-induced acidification. *L*_mix_’s higher cumulative efflux underscores the role of residue chemistry (e.g., high N, low lignin) in driving CO_2_ release, consistent with faster decomposition rates and greater H^+^ production.^26,34,57^

These results underscore a critical yet often overlooked dimension of soil–atmosphere carbon exchange. The transient but substantial contribution of SIC to CO_2_ efflux—up to 60% during early decomposition—highlights the necessity of incorporating inorganic C dynamics into terrestrial carbon models, particularly in calcareous and semiarid soils. Failing to account for SIC fluxes may lead to significant underestimations of ecosystem carbon losses during periods of active residue turnover.

In summary, soil inorganic carbon (SIC) contributes to soil CO_2_ dynamics, particularly during residue decomposition. However, a portion of the CO_2_ emitted from carbonate dissolution can be recompensated through secondary carbonate precipitation at deeper soil layers or may remain as bicarbonate ions in solution (Figure 7). The magnitude and timing of SIC-derived CO_2_ fluxes are tightly controlled by the residue quality and placement strategy. While these processes buffer the net inorganic CO_2_ loss, they exert indirect effects on soil organic carbon (SOC) stability. The depletion of carbonates from the topsoil reduces mineral protection and destabilizes SOC, thereby enhancing its vulnerability to microbial decomposition and subsequent CO_2_ emission. This coupling between SIC depletion and SOC destabilization underscores the need for balanced residue-fertilization management to preserve soil inorganic carbon stocks and mitigate CO_2_ emissions (Figure 4). By integrating high-resolution δ^13^C tracing with spatiotemporal monitoring, this study provides mechanistic insight into carbon transformations at the soil–-atmosphere interface and refines the representation of inorganic–organic carbon linkages in biogeochemical models.

Although this incubation study was conducted under controlled laboratory conditions (25 °C, 80% soil–water saturation) using short (10 cm) repacked soil columns, such settings simplify the complexity of field environments. In natural systems, fluctuating moisture, temperature gradients, and root–microbe interactions can alter the CO_2_ diffusion, carbonate reactivity, and acid–base buffering throughout the profile. For instance, rainfall events, root respiration pulses, or (more frequent) drying–rewetting cycles can transiently raise soil pCO_2_ and trigger localized carbonate dissolution. Therefore, the observed acidification and calcite dissolution rates likely represent upper-bound responses under stable, well-mixed conditions. Field-scale validations under variable hydrological and biological regimes are needed to constrain these dynamics.

Despite these constraints, the findings have important implications for soil carbon management. The demonstrated coupling between residue-derived biogenic CO_2_ and SIC dissolution highlights an overlooked feedback that can offset soil organic carbon gains. Management practices (such as no-till systems) that promote residue additions, enhance residue–soil contact, or accelerate decomposition can inadvertently intensify inorganic C loss. Incorporating SIC turnover into soil carbon models and greenhouse gas inventories will improve predictions of land–atmosphere CO_2_ exchange and strengthen the design of sustainable carbon mitigation strategies.

## Supplementary Material



## Notes

Additional equations for water flow, solute transport, and calcite dissolution models detailing HYDRUS-PHREEQC simulations; pH statistical analyses (codes available at P2KPTechSolutionsHub/Statistical-Analyses); and supplementary tables (soil texture, SIC stocks) and figures (cation exchange capacity, replica pH heatmaps, and ionic fluxes) (PDF)
