# Supplementary material for: Modeling Calcite’s Sensitivity to Biogenic CO2 Production: A Pathway to Soil CO2 Efflux Partitioning
Source: Environ Sci Technol. 2025 Oct 30;59(44):23869–82. doi: 10.1021/acs.est.5c07428 (PMC12613815; doi:10.1021/acs.est.5c07428)
Supplement: Supplementary file 1 [file es5c07428_si_001.pdf]

Supporting information for the manuscript:

Modelling Calcite's Sensitivity to Biogenic CO_2 Production: A Pathway to Soil CO_2 Efflux Partitioning

Kenneth Tetteh[1*], Georg Guggenberger[1], Sauheitl, Leopold[1], Kazem Zamanian[1*]

[1.] Institute of Earth System Sciences, Section Soil Science, Leibniz University Hannover, Herrenhäuser Straße 2, 30419, Hannover, Germany

* Corresponding authors: tetteh@ifbk.uni-hannover.de, zamanians@yahoo.com

This file includes:

- Number of pages: 11
- Number of figures: 3
- Number of tables: 5
- Number of Equations: 11

Contents: Page

- S1. Water flow models (equation (Eq. S1-S3) S1
- Solute transport & calcite dissolution models (Eq. S4–S10) S2
- Table S1: Clay, silt, and sand fractions of soil material S3
- Carbon Stocks Calculation and Statistical Analyses S4
- Figure S1: Soil cation exchange capacity and base saturation S4
- Figure S2: Spatiotemporal visualization of calcite reactivity using RGB-to-pH heatmaps. S5-S6
- Table S3: Layer-resolved soil pH with statistical indicators S7
- S2.3 Carbon Balance: Analytical vs. simulation SIC Stocks S7-S8
- Tables S4-S5: SIC stocks across profiles S9
- Figure S3: Electrical balance & ionic fluxes due to carbonate dissolution S10
- References S11

S1. MATERIALS AND METHOD

S1.1. Experimental Constraints and Dissolution Modelling

Water flow was modeled by solving the Richards equation:

$$\frac{\partial\theta(h)}{\partial t} = \frac{\partial}{\partial z}\left[K(h)\left(\frac{\partial h}{\partial z} + cos\gamma\right)\right] - S(h) \qquad \text{(S1)}$$

where θ represents the volumetric water content ($cm^3\ cm^{-3}$), h is the matric potential head (cm), t is time (d), K is the unsaturated hydraulic conductivity function ($cm\ d^{-1}$), z is a depth coordinate (negative downward) (cm), γ is the angle of flow compared to the vertical axis (i.e., $\gamma = 0°$ for vertical flow, 90° for horizontal flow), and S is a sink term, for instance, root water uptake ($cm^3\ cm^{-3}\ d^{-1}$) [S1] which is not considered in this study.

The relationships of *θ(h)* and *K(h)* in eq S1 were described by the closed-form equation of van Genuchten[S2] and the capillary model of Mualem[S3], respectively:

$$\theta(h) = S_e(h)\ [(\theta_s - \theta_r) + \theta_r] \qquad \text{(S2)}$$

$$K(h) = K_s S_e(h)^{0.5}\left[1 - (1 - S_e^{\frac{1}{m}})^m\right]^2 \qquad \text{(S3)}$$

where the degree of water content saturation, $S_e(h) = \left[\frac{1}{1+(\alpha|h|)^n}\right]^m$, θ_r and θ_s denote the residual and saturated soil water content ($cm^3\ cm^{-3}$), respectively, and n (−), m (−) and α (cm^{-1}) are empirical parameters. In this study, m was constrained as $1 - 1/n$.

Solute transport was calculated by resolving the partial differential equation governing one-dimensional convection and advection-dispersion transport models [S4]:

$$\frac{\partial\theta C_T}{\partial t} + \rho\frac{\partial\theta \underline{C}_T}{\partial t} + \rho\frac{\partial\theta \hat{C}_T}{\partial t} = \frac{\partial}{\partial z}\left[\theta D_e \frac{\partial C_T}{\partial z} - qC_T\right] \qquad \text{(S4)}$$

where C_T is the total dissolved solute concentrations (g cm^3), $\underline{C}_T$ is the total adsorbed solute concentration of the aqueous (g g^{-1} soil), $\hat{C}_T$, is the non-adsorbed solid phase concentration of aqueous solutes (g g^{-1} soil), ρ is the bulk density of the silty loam soil (g cm^{-3}), D_e is the dispersion coefficient (cm^2 d^{-1}), q is the volumetric flux density (cm d^{-1}), and n_s is the number of aqueous components resolved to be 4 for transport of carbonate reactive species: Ca^{2+}, H, C, and O. The coefficient D_e is the sum of the diffusion and dispersion components:

$$D_e = \tau D_m + \lambda \frac{|q|}{\theta} \tag{S5}$$

where τ is the tortuosity factor (-), D_m is the coefficient of molecular diffusion (cm^2 d^{-1}), and λ is the longitudinal dispersivity (cm). The presented water flow and solute transport equations were solved using the Galerkin finite element method. [S5]

Calcite dissolution is described by three parallel reactions involving proton (H^+), carbonic acid (H_2CO_3'), and water (H_2O), [S6, S7] where H_2CO_3 represents the sum of both aqueous CO_2 and H_2CO_3 ($[H_2CO_3'] = ([CO_2\,(aq)] + [H_2CO_3])$. These reactions are

$$CaCO_3 + H^+ \rightleftharpoons Ca^{2+} + HCO_3^- \tag{S6}$$

$$CaCO_3 + H_2CO_3' \rightleftharpoons Ca^{2+} + 2HCO_3^- \tag{S7}$$

$$CaCO_3 + H_2O \rightleftharpoons Ca^{2+} + HCO_3^- + OH \tag{S8}$$

The overall reaction in the H_2O–CO_2–$CaCO_3$ system is expressed as

$$CaCO_3 + CO_2 + H_2O \rightleftharpoons Ca^{2+} + 2HCO_3^- \tag{S9}$$

The net rate of dissolution for reactions S7–S9 is expressed as

$$r = k_1 \alpha_{H^+} + k_2 \alpha_{H_2CO_3'} + k_3 \alpha_{H_2O} - k_4 \alpha_{Ca^{2+}} . \alpha_{HCO_3^-}$$

(S10)

Where k_1, k_2, and k_3 are temperature-dependent first-order rate constants and k_4 is a temperature-dependent second-order rate constant. [S6] An isothermal condition of 25°C was considered. The relative contribution of each reactant is dependent on the chemical environment, specifically, on pH and soil pCO_2. At low pH (<5) and low soil p CO_2 H^+ activity dominates (first term of Eq S11) reflecting far equilibrium conditions. At sufficiently low pH (5-6.5) and high soil pCO_2, H_2CO_3' activity dominates (second term of the of Eq S11). At circumneutral pH and low soil pCO_2, H_2O activity dominates (third term of Eq S11) as H^+ activity become irrelevant. Close to equilibrium, precipitation may occur (fourth term), though this was excluded in the forward-biased dissolution model.

Soil respiration influences soil pCO_2 dynamics, which, in turn, regulates equilibrium conditions. The effects of residue quality (Tables S1, S2) and native SOC (Table 1) further modulate this process.

Table S1 Clay, silt, and sand fraction of soil material.

Material	Clay (%)	Silt (%)	Sand (%)	Textural class
Soil	22.5	68.3	9.2	Silt loam

S1.2. Carbon Stocks in SIC Pool

To quantify the export rate of bicarbonates, the carbon stocks in soil inorganic carbon (SIC) resulting from carbonate decomposition were calculated using Eq. S12:

$$\boldsymbol{C_{SIC} = \sum_{i=1}^{n} C_{SIC,i} \times \rho \times d} \qquad \text{(S11)}$$

Where C_{SIC}, represents the total SIC carbon stock (t ha^{-1}) exported across the 0–10 cm soil depth. The subscript i represents the i-th depth increment (0−1, 1−2, ..., 9−10 cm), superscript n is the total number of depth intervals. C_{SIC} is the carbon contents (g kg^{-1}) of exported SIC. ρ

is soil bulk density (kgm^{-3}), and d is the thickness of each depth increment with a uniform SIC export rate.

S1.3 Statistical analyses

The statistical analysis involved Welch's t-tests and Mann–Whitney U tests to assess pH differences across treatments, depths, and time points. Shapiro-Wilk tests were used to evaluate the normality of residuals, guiding the application of data transformations such as log, square root, and Box-Cox. This approach ensured robustness by addressing violations of parametric assumptions and validating the appropriateness of the methods used. All scripts used for the analyses are available at P2KPTechSolutionsHub/Statistical-Analyses.

S2. RESULTS

S2.1. Calcite's Sensitivity to Elevated Biogenic CO_2 Production

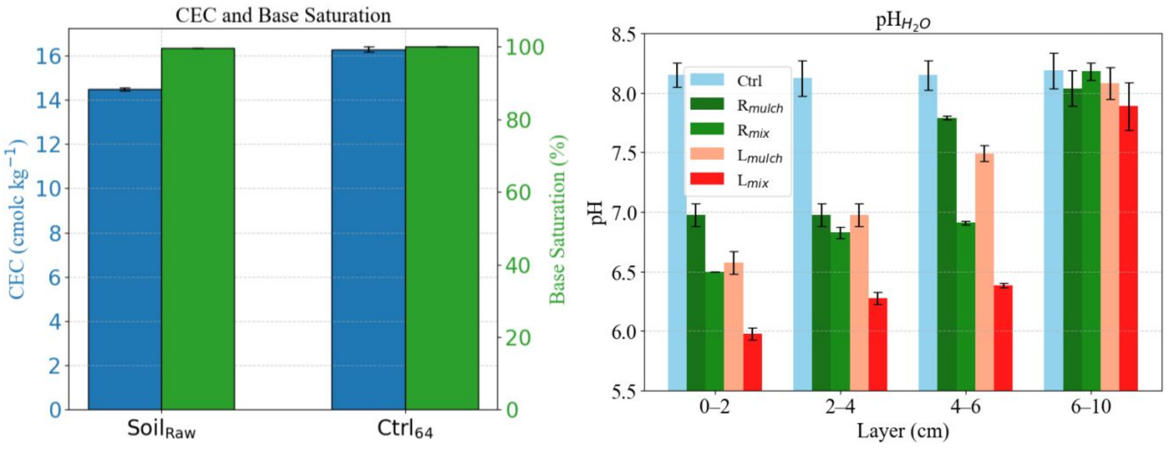

Figure S1 (a) Cation exchange capacity (CEC, blue bars) and base saturation (green bars) of raw soil and limed soil at the end of incubation (Ctrl $_{64}$). (b) Average layer-wise (amended) soil pH measured in a 1:1 (water:soil, w/w) suspension after incubation.

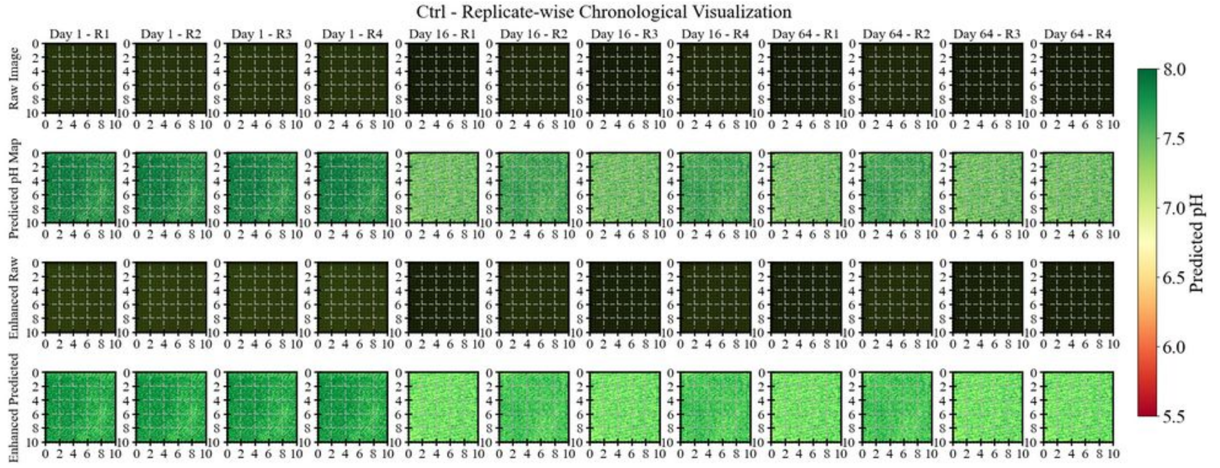
Ctrl - Replicate-wise Chronological Visualization
Day 1 - R1
Day 1 - R2
Day 1 - R3
Day 1 - R4
Day 16 - R1
Day 16 - R2
Day 16 - R3
Day 16 - R4
Day 64 - R1
Day 64 - R2
Day 64 - R3
Day 64 - R4
Raw Image
Predicted pH Map
Enhanced Raw
Enhanced Predicted
Predicted pH
8.0
7.5
7.0
6.5
6.0
5.5

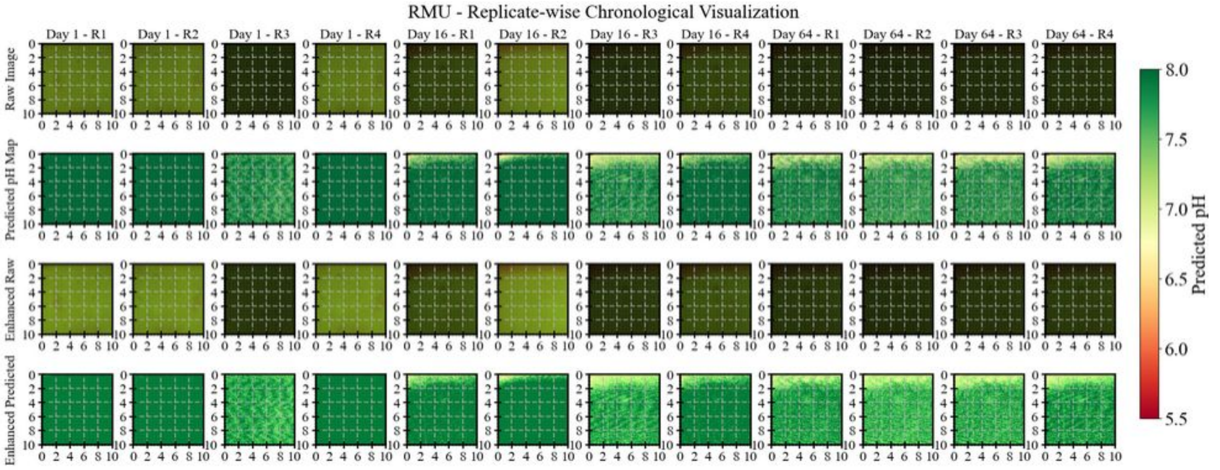
RMU - Replicate-wise Chronological Visualization
Day 1 - R1
Day 1 - R2
Day 1 - R3
Day 1 - R4
Day 16 - R1
Day 16 - R2
Day 16 - R3
Day 16 - R4
Day 64 - R1
Day 64 - R2
Day 64 - R3
Day 64 - R4
Raw Image
Predicted pH Map
Enhanced Raw
Enhanced Predicted
Predicted pH
8.0
7.5
7.0
6.5
6.0
5.5

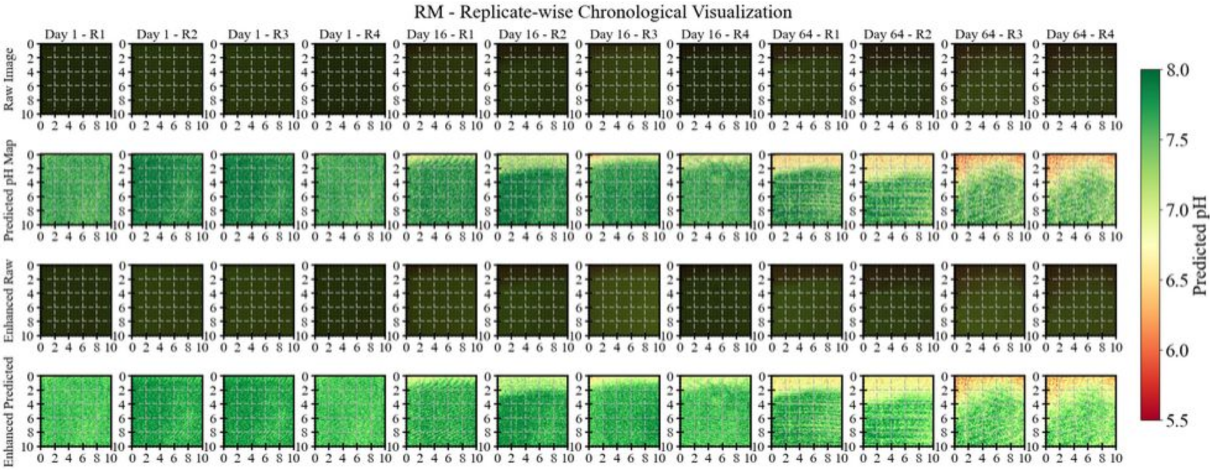
RM - Replicate-wise Chronological Visualization
Day 1 - R1
Day 1 - R2
Day 1 - R3
Day 1 - R4
Day 16 - R1
Day 16 - R2
Day 16 - R3
Day 16 - R4
Day 64 - R1
Day 64 - R2
Day 64 - R3
Day 64 - R4
Raw Image
Predicted pH Map
Enhanced Raw
Enhanced Predicted
Predicted pH
8.0
7.5
7.0
6.5
6.0
5.5

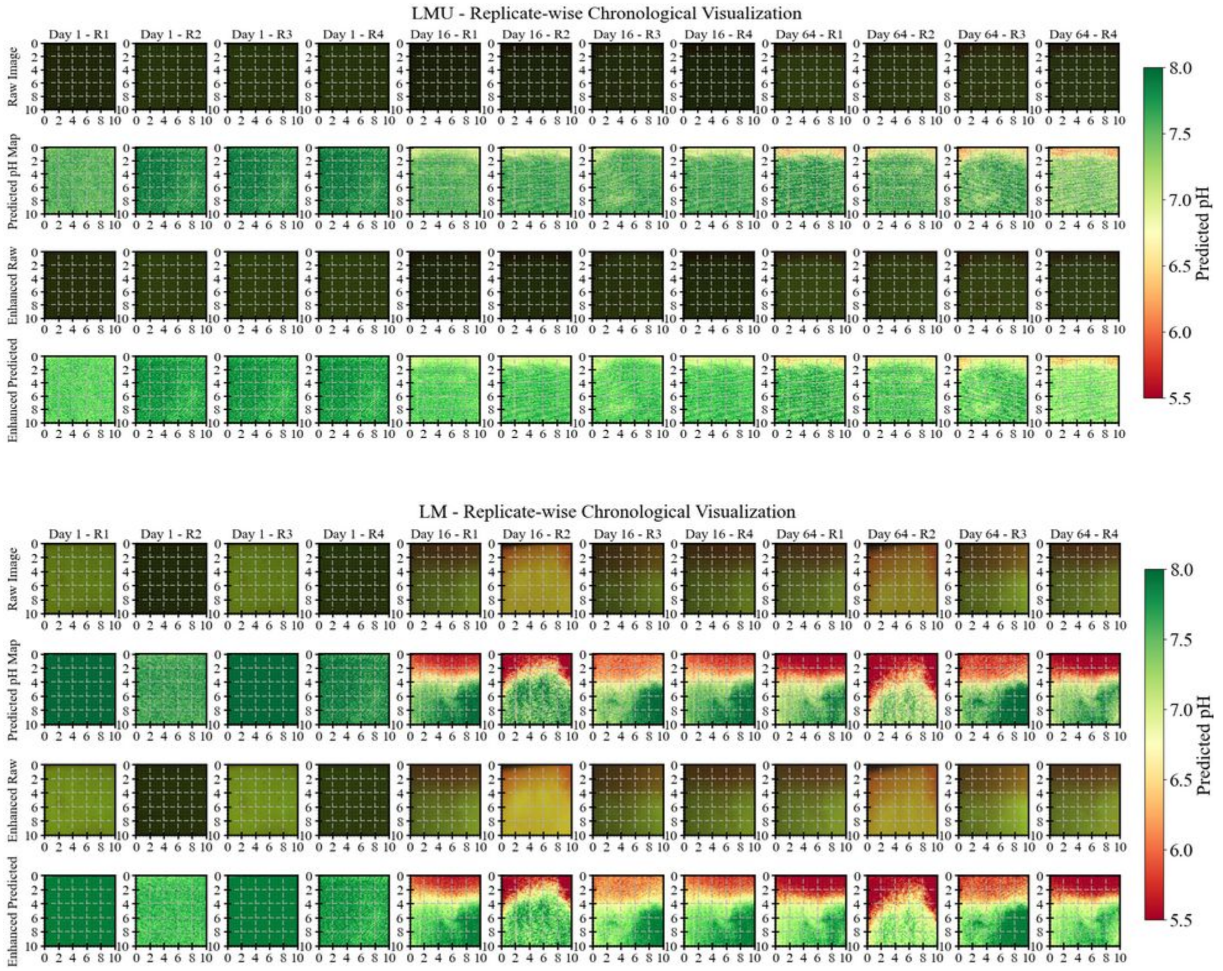

Figure. S2 Spatiotemporal visualization of calcite reactivity following decomposition across experimental treatments. For each treatment group (Ctrl, RMU, RM, LMU, LM), representative replicate images were sampled at days 1, 16, and 64. The four rows correspond to: (i) raw optode images, (ii) predicted pH heatmaps generated by a trained RGB-to-pH deep learning model, (iii) brightness-enhanced raw images, and (iv) contrast-enhanced pH prediction (for improved visualization). Temporal progression across replicates reveals treatment-specific acidification trajectories and buffering responses.

S2.2. Statistical Analysis

Table S3 Soil pH levels measured at different depths (0–5 cm) on Days 16 and 64 under four treatments: Root Mulched, Root Mixed, Leaf Mulched, and Leaf Mixed. Small letters (a, b, c) indicate significant differences among depths within the same treatment. Capital letters (A, B,

CDH) indicate sign ificant differences among treatments at the same depth. Additional small letters (x, y, z) denote significant differences across days within the same depth and treatment.

Depth (cm)	R_{mulch} (Day 16)	R_{mulch} (Day 64)	R_{mix} (Day 16)	R_{mix} (Day 64)	L_{mulch} (Day 16)	L_{mulch} (Day 64)	L_{mix} (Day 16)	L_{mix} (Day 64)
0	6.5 (bDx)	6.4(aDx)	6.4(bCx)	6.0(Hx)	6.2(bBx)	6.0 (Hx)	5.8(aAx)	5.7 (aAx)
1	6.5 (kDx)	6.4 (aIx)	6.4(kCx)	6.3(Hx)	6.2 (kB)	6.0 (Hx)	5.8(aAx)	5.7 (aAx)
2	6.5 (kD)	7.2 (uD)	6.4(kCx)	6.4(Hx)	6.2 (kB)	6.5 (H)	5.9 (Ax)	5.7 (Ax)
3	7.2 (Dx)	7.2 (Dx)	6.5 (H)	7.0 (H)	7.1 (Hx)	6.7 (H)	6.0 (Ax)	5.8 (aAx)
4	7.8 (x)	7.8 (x)	7.2 (Hx)	7.0 (x)	7.2 (Hx)	7.2 (Hx)	6.2 (A)	6.0 (Ax)
5	7.8 (x)	7.8 (x)	7.8 (x)	7.8 (x)	7.9 (x)	7.9 (x)	7.0 (Ax)	6.0 (Ax)

S2.3. Carbon Balance Computation: Analytical vs. Simulation

To rigorously quantify soil carbon stocks and validate model-derived carbon fluxes, we employed a carbon balance approach, cross-referencing analytical calculations with simulation outputs. The initial application of 1g $CaCO_3$ powder to 99 g of soil (bulk density: 1.19 g cm^{-3}) in both laboratory and numerical experiments defines the total inorganic carbon (TIC) pool. This corresponds to an applied 11.90 t ha^{-1} of $CaCO_3$, equivalent to 1.428 t C ha^{-1}, based on a molar mass of 100.08 g mol^{-1}. Given a homogeneous distribution, 50% of the applied $CaCO_3$ resides at or below the 5 cm depth, yielding a carbonate stock of 0.714 t C ha^{-1} at mid-profile.

Far from equilibrium, complete $CaCO_3$ dissolution was observed, facilitating bicarbonate export throughout the 0–5 cm depth profile (Figure 5e, 6e, 7e). The simulation outputs computed total dissolved inorganic carbon (DIC) at 0.836 t C ha^{-1} (Table S4). This DIC pool integrates both direct carbonate dissolution and biogenic CO_2-driven bicarbonate formation, governed by carbonate equilibria (Eq. 2–3). The elevated soil pCO_2, induced by microbial respiration and organic residue decomposition, actively promoted carbonate dissolution. Of the total DIC generated, 15.07% (0.126 t C ha^{-1}) originated from surplus biogenic CO_2, which

transformed into bicarbonate and was subsequently exported, while 84.93% (0.714 t C ha^{-1}) derived directly from the applied calcite powder.

Notably, an even greater fraction of the boundary pCO_2 fuels $CaCO_3$ dissolution, underscoring its role in driving carbonate equilibrium shifts. This direct consumption of biogenic CO_2 in mineral dissolution represents an active inorganic sink for atmospheric carbon, linking microbial respiration and residue degradation to carbonate decomposition. While this process disrupts soil alkalinity, it sequesters/ stabilizes carbon through bicarbonate export and potential reprecipitation as secondary carbonates.

These findings highlight the regulatory role of the time-variable boundary soil pCO_2 in carbonate transformations, reinforcing the dynamic continuum between soil inorganic carbon and biogenic carbon fluxes. The model captured these processes, with no evidence of carbon flux underestimation (Table S4, S5).

Beyond short-term dissolution dynamics, our results underscore the significance of soil carbonate pools as transient and long-term carbon sinks. Inorganic carbon sequestration occurs via bicarbonate export to deeper layers, where it may contribute to secondary carbonate precipitation or fluvial transport, key mechanisms in the global carbon cycle. Concurrently, organic amendments sustain biogenic C sinks, driving microbial carbon stabilization and occlusion within soil aggregates. By integrating numerical simulation with mass balance constraints, this study provides new insights into coupled carbonate dissolution pathways, bicarbonate export dynamics, and soil C flux computations, reinforcing the accuracy of modeled carbon transformations in heterogeneous soil systems.

Table S4 SIC stocks exported across soil layers following calcite dissolution under mixed-leaf profile.

Depth (cm)	Molar Concentration (mol/1000 cm³)	C per kg soil (g kg^{-1})	C Stock (t C ha^{-1})
0-2	0.002	0.0202	0.481
2-3	0.00123	0.0124	0.148
3-5	(0.00123-0.00050)	0.0087	0.207

Table S5 SIC stocks exported across soil layers following calcite dissolution under mulched-leaf profile.

Depth (cm)	Molar Concentration (mol/1000 cm³)	C per kg soil (g kg^{-1})	C Stock (t C ha^{-1})
0	0.00052	0.00525	-
1	0.00040	0.00404	-
0-1	(0.00052-0.00040)	0.00465	0.0552

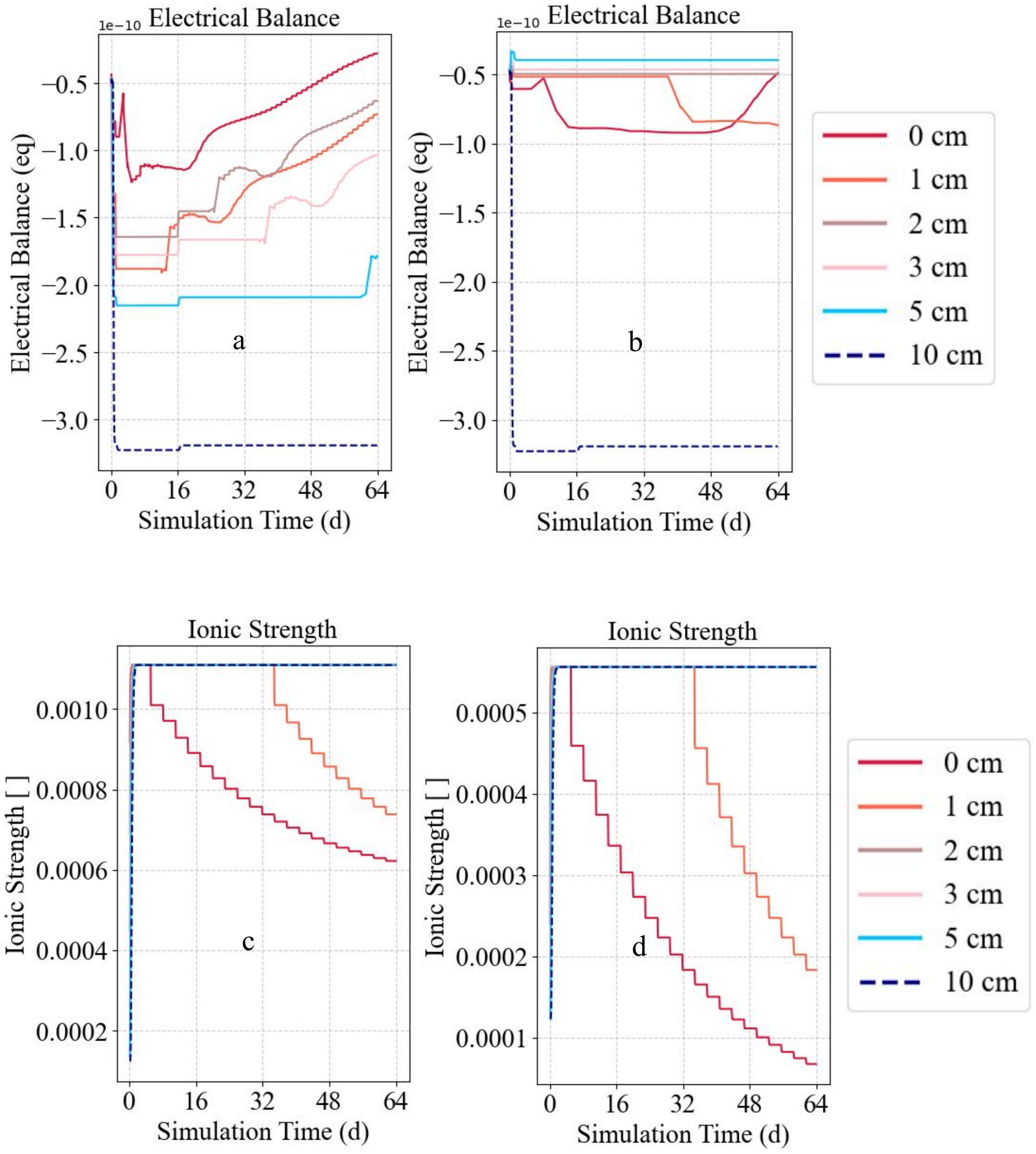

Figure S3. Electric balance trends (top), highlighting stable electronegativity in the subsoil and imbalances at the surface due to elevated pCO_2, which induces calcite dissolution and export of H^+ and bicarbonate ions. This export drives a decline in ionic strength within the top layer, creating a dynamic surface-layer ionic flux. A stabilizing ionic gradient is observed in deeper soil horizons, where reduced microbial activity and carbonate flux maintain near-equilibrium ionic concentrations. Left: mixed amendment profile; Right: mulched amendment profile.

REFERENCES

S1. Šimůnek, J.; Jacques, D.; Twarakavi, N. K. C.; van Genuchten, M. Th. Modeling Subsurface Flow and Contaminant Transport as Influenced by Biological Processes at Various Scales Using Selected HYDRUS Modules. *Biologia* 2009, *64* (3), 465–469. https://doi.org/10.2478/s11756-009-0106-7.

S2. van Genuchten, M. T. A closed-form equation for predicting the hydraulic conductivity of unsaturated soils. *Soil Sci. Soc. Am. J.* 1980, 44, 892–898. https://doi.org/10.2136/sssaj1980.03615995004400050002x.

S3. Mualem, Y. A New Model for Predicting the Hydraulic Conductivity of Unsaturated Porous Media. *Water Resour. Res.* 1976, *12* (3), 513–522. https://doi.org/10.1029/WR012i003p00513.

S4. Persicani, D. Pesticide leaching into field soils: Sensitivity analysis of four mathematical models. *Ecol. Model.* 1996, 84(1-3), 265-280. https://doi.org/10.1016/0304-3800(94)00136-7

S5. Šimůnek, J.; Suarez, D. L. Modeling of Carbon Dioxide Transport and Production in Soil: 1. Model Development. *Water Resour. Res.* 1993, *29* (2), 487–497. https://doi.org/10.1029/92WR02225.

S6. Plummer, L. N.; Wigley, T. M. L.; Parkhurst, D. L. The Kinetics of Calcite Dissolution in CO_2-Water Systems at 5 °C to 60 °C.

S7. Chou, L.; Garrels, R. M.; Wollast, R. Comparative Study of the Kinetics and Mechanisms of Dissolution of Carbonate Minerals. *Chem. Geol.* 1989, *78* (3−4), 269−282.
